# Taxonomic notes on the armored spiders of the families Pacullidae and Tetrablemmidae (Arachnida, Araneae) from Singapore

**DOI:** 10.3897/zookeys.661.10677

**Published:** 2017-03-14

**Authors:** Yucheng Lin, Joseph K. H. Koh, Seppo Koponen, Shuqiang Li

**Affiliations:** 1 Key Laboratory of Bio-resources and Eco-environment (Ministry of Education), College of Life Sciences, Sichuan University, Chengdu, Sichuan 610064, China; 2 Southeast Asia Biodiversity Research Institute, Chinese Academy of Sciences, Yezin, Nay Pyi Taw 05282, Myanmar; 3 National Biodiversity Centre, National Parks Board, Singapore 259569; 4 Zoological Museum, Biodiversity Unit, FI-20014 University of Turku, Finland; 5 Institute of Zoology, Chinese Academy of Sciences, Beijing 100101, China; 6 University of Chinese Academy of Sciences, Beijing 100049, China; 7 Shuqiang Li

**Keywords:** Taxonomy, pacullids, tetrablemmids, morphology, southeast Asia

## Abstract

Eight species of armored spiders belonging to two families, Pacullidae Simon, 1894 and Tetrablemmidae O. Pickard-Cambridge, 1873, are reported from Singapore. Five species are documented as new to science: *Paculla bukittimahensis* Lin & Li, **sp. n.** (male and female), *Paculla globosa* Lin & Li, **sp. n.** (male and female), *Ablemma malacca* Lin & Li, **sp. n.** (male and female), *Singaporemma lenachanae* Lin & Li, **sp. n.** (male and female), and *Sulaimania brevis* Lin & Li, **sp. n.** (male). The three known species are *Brignoliella besutensis* Lin, Li & Jäger, 2012, *Brignoliella michaeli* Lehtinen, 1981, and *Singaporemma singulare* Shear, 1978, of which the female of *Brignoliella besutensis* is described for the first time. For comparison, types of *Singaporemma adjacens* Lehtinen, 1981 from Vietnam, *Singaporemma halongense* Lehtinen, 1981 from Vietnam, *Singaporemma singulare* from Singapore and *Sulaimania vigelandi* Lehtinen, 1981 from Malaysia are studied and photographed.

## Introduction

Tetrablemmids and pacullids are collectively known as armored spiders because their abdomen is characteristically armor-plated with complicated abdominal scuta. Family placement of these haplogyne spiders has gone through a rather tortuous journey. Tetrablemmidae was first established by O. Pickard-Cambridge (1873) with *Tetrablemma* as its type genus. The family name Pacullidae was first used by Thorell (1898). He had taken the cue from Simon (1889, 1894) who had replaced the preoccupied name *Phaedima* in (Thorell 1881) with *Paculla*, and grouped it, along with *Perania* and *Tetrablemma* in Paculleae, but under the family Theridiidae. Roewer (1963) placed *Paculla*, *Tetrablemma*, and a few other tetrablemmids under another family, viz., Hadrotarsidae. The family placements of these armored spiders were negated when Levi and Levi (1962) and Levi (1968) transferred them out of Theridiidae and Hadrotarsidae respectively. Believing that they were monophyletic, Brignoli (1973) subsumed Pacullidae (*sensu* Thorell) under Tetrablemmidae. In an extensive survey of the armored spiders, Shear (1978) pointed out that some of the *Paculla* described by Simon, Roewer and Brignoli were not the *Paculla* (*sensu Phaedima*) as initially conceived by Thorell (1898). More significantly, while agreeing that Pacullidae and Tetrablemmidae were closely related, he argued that more study was needed before the two families were combined. Nevertheless, Lehtinen (1981) decided to incorporate Pacullidae, then with a sole genus *Paculla*, as a sub-family under Tetrablemmidae. More recently, however, based on target-gene analyses from extensive spider taxa, Wheeler et al. (2016) have restored the family status of Pacullidae and circumscribed Tetrablemmidae, with redefined diagnoses and composition. Here, their family placement in reporting the armored spiders from Singapore are adopted.

According to Murphy and Murphy (2000: 548, fig 59.1–3), Singapore was home to four species of armored spiders. They included *Singaporemma singulare* Shear, 1978, which was the type species of the genus named after Singapore. The remaining three armored spiders comprised an unidentified species of *Paculla* collected from Bukit Timah in Singapore, and two species previously described from Malaysia and Vietnam: *Brignoliella michaeli* Lehtinen, 1981 and *Singaporemma halongense* Lehtinen, 1981. Our current study suggests that the *Singaporemma* in Singapore comprise *Singaporemma singulare* and a second *Singaporemma* not identical to *Singaporemma halongense*, but a new species closely related to it. This study further suggests that the unidentified *Paculla* referred to by Murphy and Murphy (2000: 370, fig 59.1) is probably one of the two new species of *Paculla* described in this paper.

Altogether, this paper records a total of eight species of armored spiders in Singapore. They include two new species of *Paculla* (Pacullidae). Among the Tetrablemmidae described and re-described in this paper are *Singaporemma singulare*, along with a new species each of *Ablemma*, *Singaporemma* and *Sulaimania*. The presence of *Brignoliella besutensis* Lin, Li & Jäger, 2012 in Singapore was ascertained for the first time, while the presence of *Brignoliella michaeli* was revalidated.

## Materials and methods

All specimens were collected from July to August 2015 from various locations in Singapore by sifting leaf litter. Specimens were preserved in 95% ethanol. They were examined and measured under a Leica M205 C stereomicroscope. Further details were studied under an Olympus BX43 compound microscope. Vulvae were removed and treated in lactic acid. To reveal the course of the spermatic duct, the palpal bulbs were treated in lactic acid and mounted in Hoyer’s Solution. Photographs were taken with a Canon EOS 60D wide zoom digital camera (8.5 megapixels). The images were combined using Helicon Focus 3.10.3 software (Khmelik et al. 2006).

All measurements are in millimetres. Height of carapace is measured with tubercle. Leg measurements are given in the following sequence: total length (femur, patella, tibia, metatarsus, and tarsus). Abbreviations in figures are as follows: **A** – anal plate; **ALG** – anterolateral groove of preanal plate; **AT** – atrium; **AV** – anterior ventrolateral plate; **EF** – epigynal fold; **EP** – epigynal pit; **ep** – embolic part of apes of palpal organ; **IVP** – inner vulval plate; **L** – lateral plate; **LH** – lateral horn; **MV** – median ventrolateral plate; **MVB** – bridge fragments of MV; **P** – pulmonary plate; **PA** – preanal plate; **PG** – postgenital plate; **PLC** – posterolateral corner of PA; **PMC** – posteromedial corner of PA; **PV** – posterior ventrolateral plate; **sd** – spermatic duct; **sl** – subterminal lamella; **SR** – seminal receptaculum; **VD** – vulval duct; **VS** – vulval stem. Abbreviations in text include: **AER** – anterior eye row; **ALE** – anterior lateral eye; **AME** – anterior median eye; **PLE** – posterior lateral eye. References to figures in the cited papers are listed in lowercase (fig. or figs); figures from this paper are noted with an initial capital (Fig. or Figs).

All types of the new species are deposited in the Lee Kong Chian Natural History Museum, National University of Singapore (**LKCNHM**). Other material used in the current work are deposited in the Natural History Museum of the Sichuan University (**NHMSU**) in Chengdu, China; the Zoological Museum of the University of Turku (**ZMUT**) in Turku, Finland and the American Museum of Natural History in New York, USA (**AMNH**).

## Taxonomy

### Family Pacullidae Simon, 1894

#### Genus Paculla

Taxon classificationAnimaliaAraneaePacullidae

Simon, 1887

##### Type species.

*Phaedima granulosa* Thorell, 1881 from New Guinea (see Lehtinen 1981).

#### Paculla bukittimahensis

Taxon classificationAnimaliaAraneaePacullidae

Lin & Li sp. n.

http://zoobank.org/E95B9FE6-2604-4D4C-BC86-C5E701064012

Figs 1 , 2 , 3

##### Type material.

**Holotype** ♂ (LKCNHM), SINGAPORE: Bukit Timah Nature Reserve, Catchment Path, altitude 107 m, 1°21'12.5"N, 103°46'50.6"E, 20 August 2015, S. Li and Y. Tong leg. **Paratypes** 1♂ and 5♀ (LKCNHM), same data as holotype.

##### Other material examined.

1♂ and 3♀ (NHMSU), SINGAPORE: Bukit Timah Nature Reserve, Catchment Path, altitude 107 m, 1°21'12.5"N, 103°46'50.6"E, 20 August 2015, S. Li and Y. Tong leg.

##### Etymology.

The specific name refers to the type locality; adjective.

##### Diagnosis.

**Figure 1.**
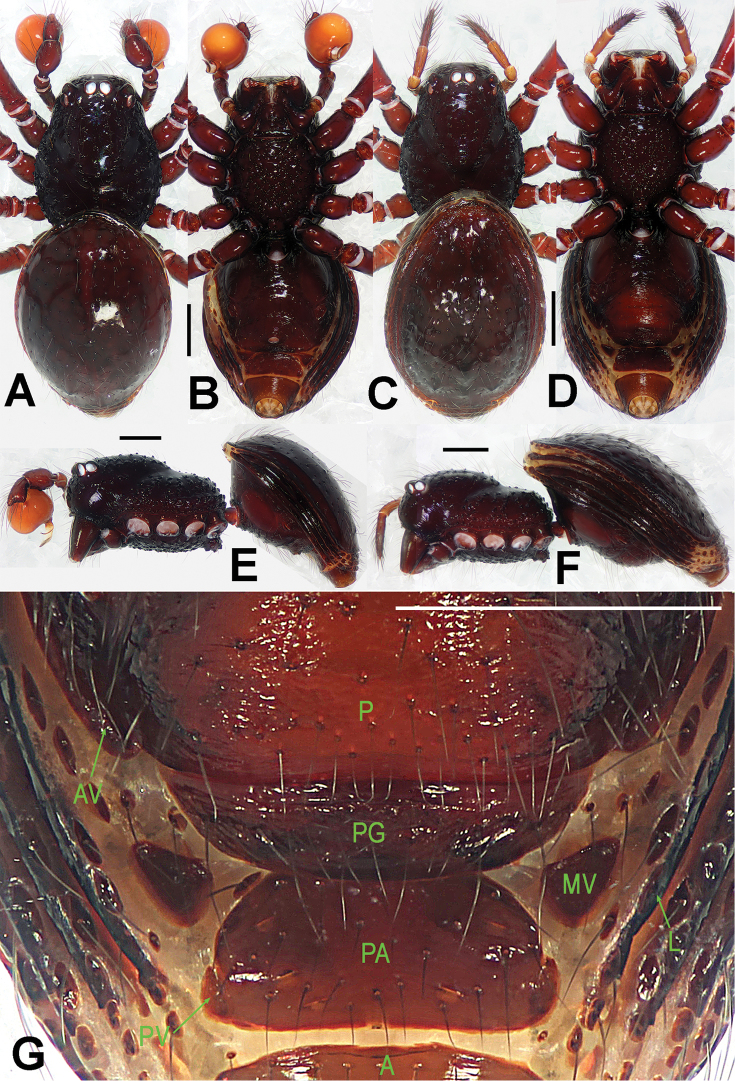
*Paculla bukittimahensis* sp. n., male holotype (**A–B, E**) and female paratype (**C–D, F–G**). **A–F** habitus **G** genital area (untreated). **A, C** dorsal **B, D, G** ventral **E–F** lateral. Abbreviations: A = anal plate; AV = anterior ventrolateral plate; L = lateral plate; P = pulmonary plate; PA = preanal plate; PG = postgenital plate; PV = posterior ventrolateral plate. Scale bars: 0.50 mm.

**Figure 2.**
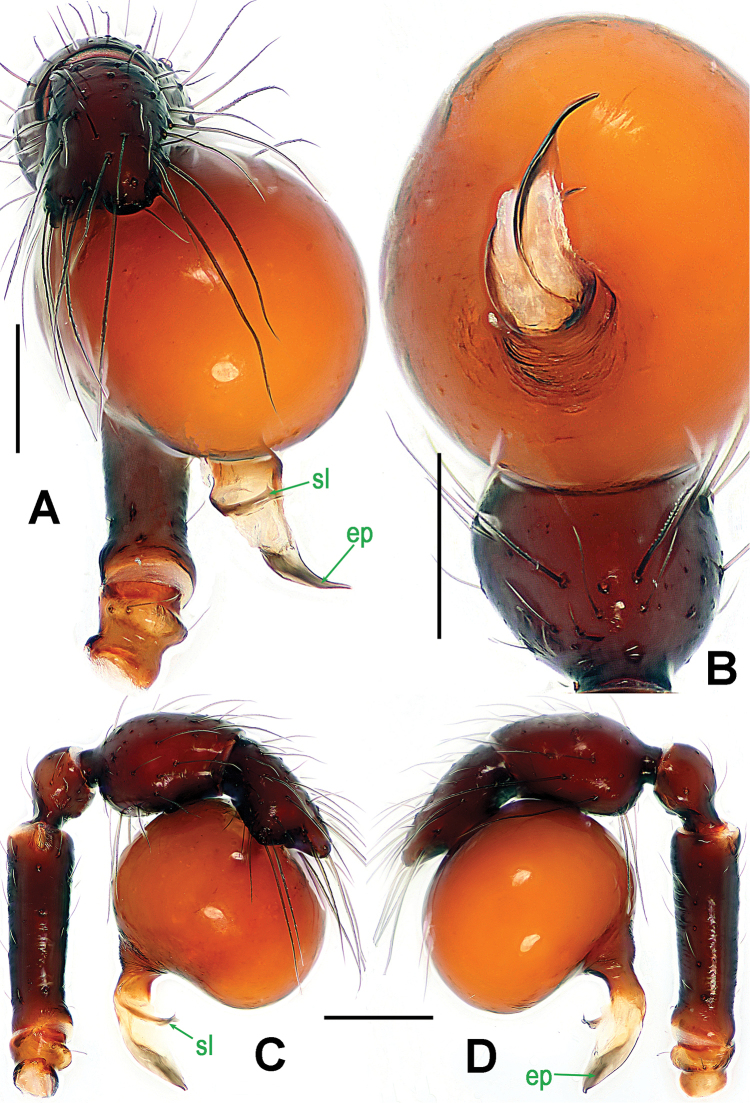
*Paculla bukittimahensis* sp. n., male holotype. **A, C–D** left palp **B** palpal bulb. **A** anterior **B** ventral **C** prolateral **D** retrolateral. Abbreviations: ep = embolic part of apes of palpal organ; sl = subterminal lamella. Scale bars: 0.20 mm.

**Figure 3.**
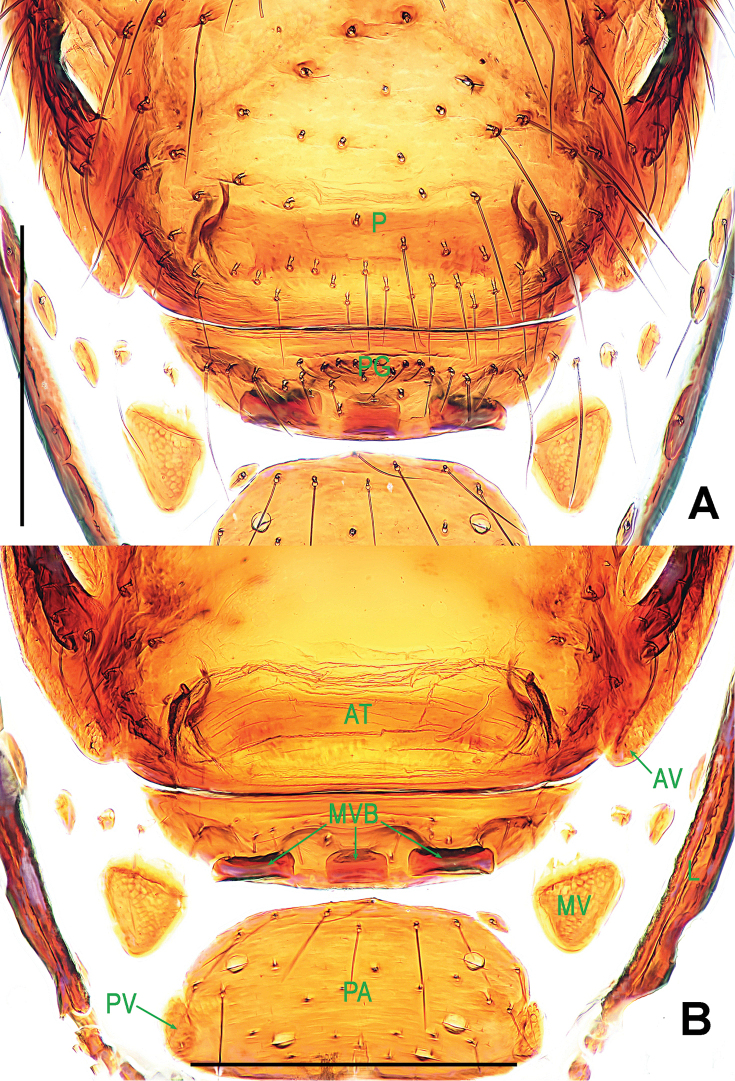
*Paculla bukittimahensis* sp. n., female paratype. **A** genital area (lactic acid-treated), ventral **B** *ditto*, dorsal. Abbreviations: AT = atrium; AV = anterior ventrolateral plate; MV = median ventrolateral plate; MVB = bridge fragments of MV; P = pulmonary plate; PA = preanal plate; PG = postgenital plate; PV = posterior ventrolateral plate. Scale bars: 0.50 mm.

This new species can be distinguished from all congeners with the exception of *Paculla mului* Bourne, 1981 and *Paculla wanlessi* Bourne, 1981 by the wide, short embolus (Fig. 2A, C–D), the well-developed postgenital scutum (Fig. 1G) and the nearly rectangular atrium (Fig. 3B). It differs from *Paculla mului* (see Bourne, 1981: 220, figs 11–17) by the normal male femur I lacking a subdistal-ventral process, the longer, particularly furcated embolus (Fig. 2A, C–D) and the presence of three disjunct bridge fragments of MV (Fig. 3B); and from *Paculla wanlessi* (see Bourne, 1981: 217, figs 1–10) by the larger bulb (Fig. 2C–D), the more pointed embolus (Fig. 2A–B), the nearly trapeziform preanal scutum (Figs 1G, 3B) and the triangular median ventrolateral plate (Figs 1G, 3A–B). It can be separated from *Paculla globosa* sp. n. (Figs 5A–D, 6A–B) by the wider, shorter embolus (Fig. 2A–B), the slightly compressed bulb (Fig. 2C–D), and the three shorter, disjunctive bridge fragments of MV (Fig. 3B) and the nearly rectangular atrium (Fig. 3B).

##### Description.

**Male** (holotype). Coloration: body dark reddish brown; legs reddish-brown.

Measurements: total length 4.05; carapace 1.80 long, 1.35 wide, 1.12 high; abdomen 2.35 long, 1.65 wide, 1.78 high; clypeus 0.45 high; sternum 1.05 long, 0.90 wide. Length of legs: I 7.01 (2.12, 0.53, 2.00, 1.52, 0.84); II 5.85 (1.81, 0.50, 1.54, 1.25, 0.75); III 4.82 (1.45, 0.45, 1.20, 1.10, 0.62); IV 6.64 (2.00, 0.51, 1.83, 1.62, 0.68).

Prosoma (Fig. 1A–B, E): carapace finely granulated, margin rugose, covered with thin setae; eyes white, ALE>AME=PLE; cephalic part moderately raised; cervical groove distinct; clypeus vertical anteriorly; labium triangular, distally obtuse; sternum rough, marginally rugose, posterior corner protruded. Legs: cuticle striated, weakly granular.

Opisthosoma (Fig. 1A–B, E): dorsal scutum long, oval, smooth, modified by tiny pits, covered with thin setae; ventral scutum rugose; lateral scutum I short, perigenital scutum triangular, postepigastral scutum same width as preanal scutum.

Palp (Fig. 2A–D): femoral cuticle slightly striated, approximately 2.5 times as long as patella; patella proximally narrow, distally wide; tibia large, swollen, 1.5 times as wide as femur; cymbium compressed, distally bifurcate; bulb tomato-shaped, surface smooth (Fig. 2C–D); embolus long, proximally sclerotized, distally rugose membranous, starting from subdistal-ventral 1/3 position of bulbous surface, and curved downwards; embolic tip flexuous, and asymmetric split ends (Fig. 2A–B).

**Female** (one of paratypes). Coloration as in male.

Measurements: total length 4.42; carapace 1.85 long, 1.40 wide, 1.13 high; abdomen 2.85 long, 1.85 wide, 2.03 high; clypeus 0.43 high; sternum 1.10 long, 0.97 wide. Length of legs: I 7.37 (2.27, 0.55, 2.10, 1.60, 0.85); II 6.11 (1.90, 0.50, 1.60, 1.38, 0.73); III 5.19 (1.55, 0.49, 1.30, 1.20, 0.65); IV 7.19 (2.15, 0.52, 2.00, 1.77, 0.75). Length of palp: 1.62 (0.48, 0.21, 0.35, 0.58).

Carapace, abdomen, and legs as in male (Fig. 1C–D, F); clypeus slightly lower than in male.

Genitalia (Figs 1G; 3A–B): epigynal area strongly sclerotized (Fig. 1G); postgenital scutum wider than preanal scutum; median ventrolateral plate triangular (Fig. 3A). Vulva with a large, nearly rectangular atrium; three bridge fragments of MV disjunctive, the medial shorter than the laterals, below the atrium, and closed to the dorso-posterior margin of postgenital scutum (Fig. 3B).

##### Distribution.

Singapore.

#### Paculla globosa

Taxon classificationAnimaliaAraneaePacullidae

Lin & Li sp. n.

http://zoobank.org/CAD62F40-4F41-4D91-8E0E-7E9AAAA1632C

Figs 4 , 5 , 6

##### Type material.

**Holotype** ♂ (LKCNHM), SINGAPORE: Bukit Timah Nature Reserve, Jungle Fall Stream, altitude 118 m, 1°21'25.4"N, 103°46'25.3"E, 21 August 2015, S. Li and Y. Tong leg. **Paratype** 1♀ (LKCNHM), SINGAPORE: Bukit Timah Nature Reserve, Bukit Timah Summit, altitude 163 m, 1°21'16.7"N, 103°46'35.0"E, 19 August 2015, S. Li and Y. Tong leg.

##### Etymology.

The specific epithet derives from the Latin word “*globosus*” = globular, drawing attention to the shape of palpal bulb; adjective.

##### Diagnosis.

This new species can be distinguished from all congeners with the exception of *Paculla sulaimani* Lehtinen, 1981 by the globular bulb (Fig. 5C–D), the long, finger-like embolus (Fig. 5A–D) and the presence of three bridge fragments of MV (Fig. 6B). Its male differs from *Paculla sulaimani* (see Lehtinen, 1981: 18, fig. 13) by the narrower embolus with weakly lobed end (Fig. 5A, C–D). The female differs from *Paculla bukittimahensis* sp. n. (Fig. 3B) by the anterior median part of the atrium protruding broadly and the larger, bridge fragments of MV almost touching each other (Fig. 6B).

**Figure 4.**
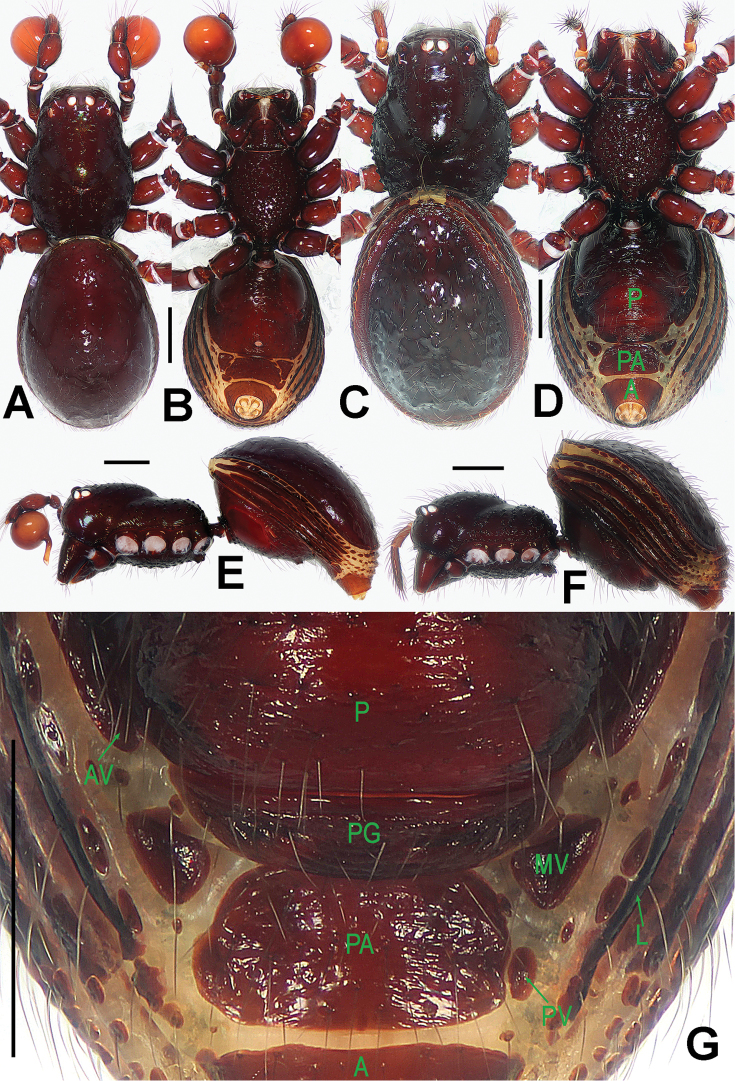
*Paculla globosa* sp. n., male holotype (**A–B, E**) and female paratype (**C–D, F–G**). **A–F** habitus **G** genital area (untreated). **A, C** dorsal **B, D, G** ventral **E–F** lateral. Abbreviations: A = anal plate; AV = anterior ventrolateral plate; L = lateral plate; MV = median ventrolateral plate; P = pulmonary plate; PA = preanal plate; PG = postgenital plate. Scale bars: 0.50 mm.

##### Description.

**Male** (holotype). Coloration: body dark reddish brown; legs reddish-brown.

Measurements: total length 4.40; carapace 1.95 long, 1.40 wide, 1.20 high; abdomen 2.50 long, 1.90 wide, 2.30 high; clypeus 0.49 high; sternum 1.30 long, 1.00 wide. Length of legs: I 8.28 (2.53, 0.65, 2.35, 1.75, 1.00); II 6.95 (2.10, 0.60, 1.85, 1.50, 0.90); III 5.75 (1.70, 0.54, 1.41, 1.30, 0.80); IV 8.16 (2.50, 0.62, 2.30, 1.91, 0.83).

Prosoma (Fig. 4A–B, E): carapace low, sparsely granulated over whole surface, but surface between the granules smooth; eyes white, ALE=AME=PLE; cephalic part moderately raised; cervical groove distinct; thoracic fovea shallow; clypeus vertical anteriorly; labium triangular, distally obtuse; sternum rough, covered with thin setae, posterior corner protruded. Legs: cuticle striated, weakly granular.

Opisthosoma (Fig. 4A–B, E): dorsal scutum long, oval, covered with thin setae, margin rugose, center modified by sparse pits; rows of small sclerites between lateral scuta, lateral scutum I short; ventral scutum smooth, margin rugose; perigenital scutum broad, triangular.

**Figure 5.**
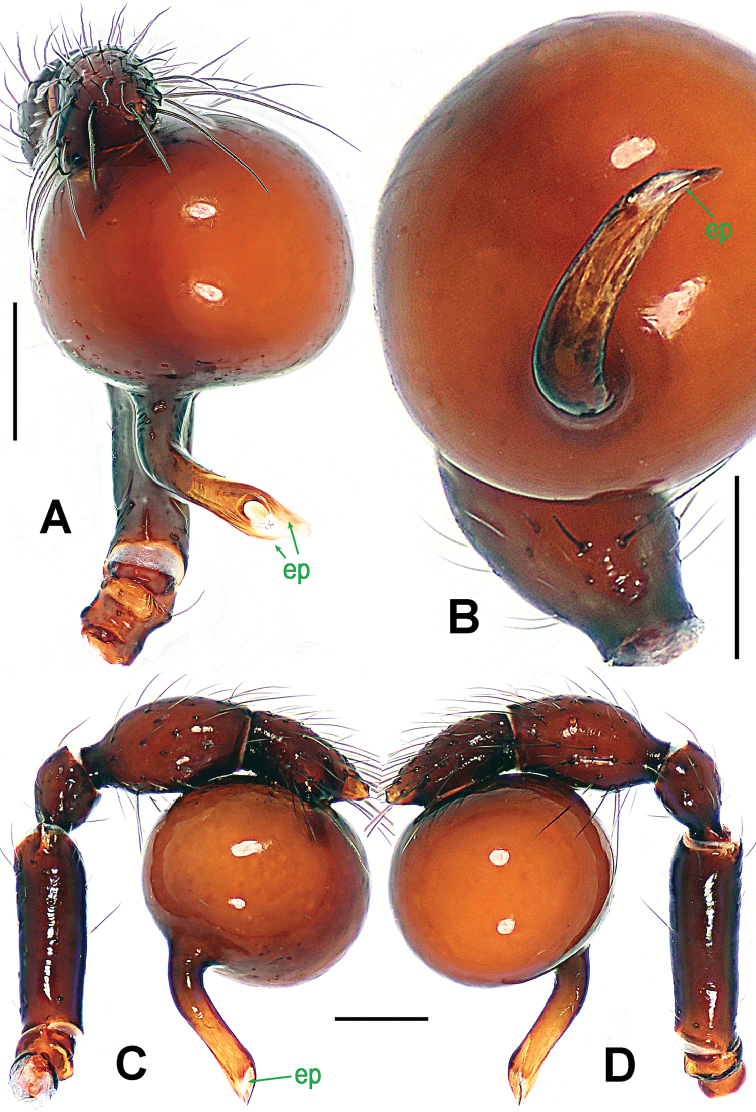
*Paculla globosa* sp. n., male holotype. **A, C–D** left palp **B** palpal bulb. **A** anterior **B** ventral **C** prolateral **D** retrolateral. Abbreviations: ep = embolic part of apes of palpal organ. Scale bars: 0.20 mm.

Palp (Fig. 5A–D): femoral cuticle smooth, approximately two times as long as patella; patella proximally narrow, distally wide; tibia broad, swollen, 1.3 times as wide as femur; cymbium compressed, slightly shorter than tibia, distally obtuse bifurcate; bulb globular, surface smooth (Fig. 5C–D); embolus long, clavate, starting from subdistal-ventral 1/3 position of bulbous surface (Fig. 5B), proximally sclerotized and bent, distally shallow furcated; embolic tip faint, with split ends (Fig. 5A).

**Female** (paratype). Coloration as in male.

Measurements: total length 4.50; carapace 1.85 long, 1.45 wide, 1.20 high; abdomen 2.67 long, 1.80 wide, 2.21 high; clypeus 0.43 high; sternum 1.14 long, 0.97 wide. Length of legs: I 7.35 (2.25, 0.55, 2.10, 1.65, 0.80); II 6.23 (1.95, 0.53, 1.65, 1.35, 0.75); III 5.22 (1.55, 0.50, 1.30, 1.22, 0.65); IV 7.20 (2.15, 0.54, 2.00, 1.80, 0.71). Length of palp: 1.58 (0.45, 0.21, 0.34, 0.38).

Carapace, abdomen, and legs as in male (Fig. 4C–D, F); clypeus slightly lower than in male.

**Figure 6.**
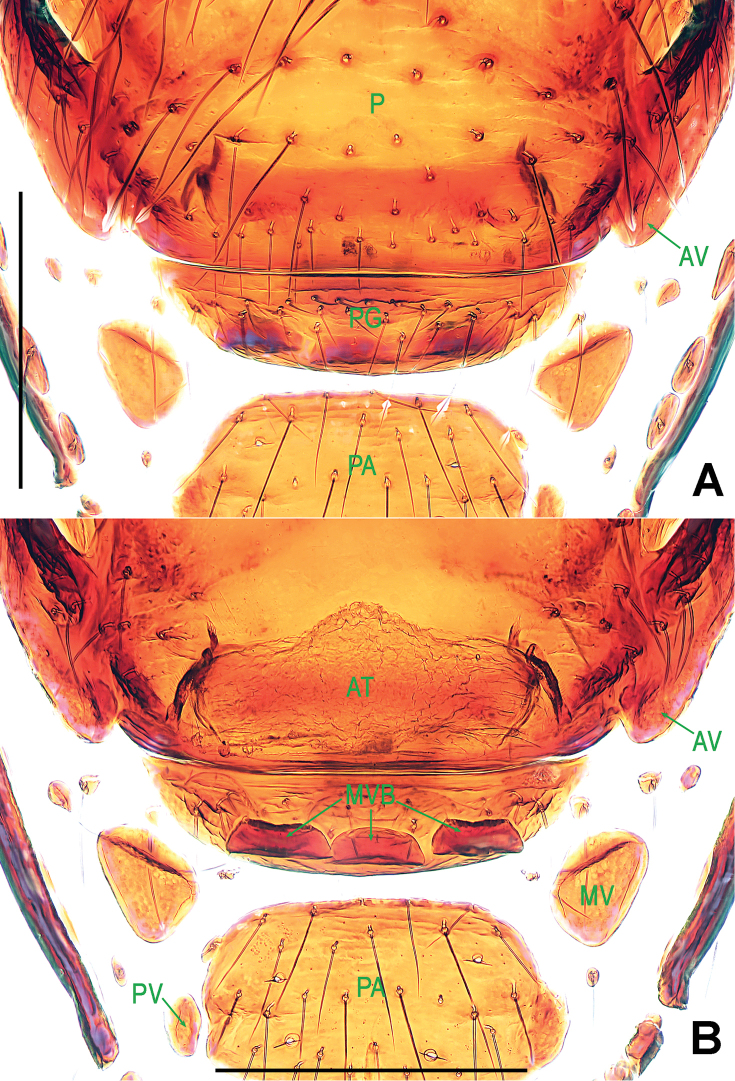
*Paculla globosa* sp. n., female paratype. **A** genital area (lactic acid-treated), ventral **B** *ditto*, dorsal. Abbreviations: AT = atrium; AV = anterior ventrolateral plate; MV = median ventrolateral plate; MVB = bridge fragments of MV; P = pulmonary plate; PA = preanal plate; PG = postgenital plate; PV = posterior ventrolateral plate. Scale bars: 0.50 mm.

Genitalia (Figs 4G; 6A–B): epigynal area strongly sclerotized (Fig. 4G); postgenital scutum rugose, wider than preanal scutum; median ventrolateral plate triangular; preanal scutum rugose, wider than long (Fig. 6A). Vulva with a large, nearly rectangular atrium, laterally rugose, and protruding anteromedially; three adjacent, equal sizes bridge fragments of MV below the atrium, and close to the dorso-posterior margin of postgenital scutum (Fig. 6B).

##### Distribution.

Singapore.

### Family Tetrablemmidae O. Pickard-Cambridge, 1873

#### Genus Ablemma

Taxon classificationAnimaliaAraneaeTetrablemmidae

Roewer, 1963

##### Type species.

*Ablemma baso* Roewer, 1963 from Sumatra (see Lehtinen 1981).

#### Ablemma malacca

Taxon classificationAnimaliaAraneaeTetrablemmidae

Lin & Li sp. n.

http://zoobank.org/4404C598-0F48-44E6-A95D-8B8A1D05DFA8

Figs 7 , 8 , 9

##### Type material.

**Holotype** ♂ (LKCNHM), SINGAPORE: Central Catchment Nature Reserve, altitude 60 m, 1°21'21.7"N, 103°48'3.8"E, 26 August 2015, S. Li and Y. Tong leg. **Paratypes** 1♂ and 2♀ (LKCNHM), same data as holotype.

##### Other material examined.

1♂ and 1♀ (NHMSU), SINGAPORE: Central Catchment Nature Reserve, altitude 60 m, 1°21'21.7"N, 103°48'3.8"E, 26 August 2015, S. Li and Y. Tong leg.

##### Etymology.

The specific epithet refers to the Strait of Malacca, which separates Singapore from the Indonesian island of Sumatra; noun.

##### Diagnosis.

**Figure 7.**
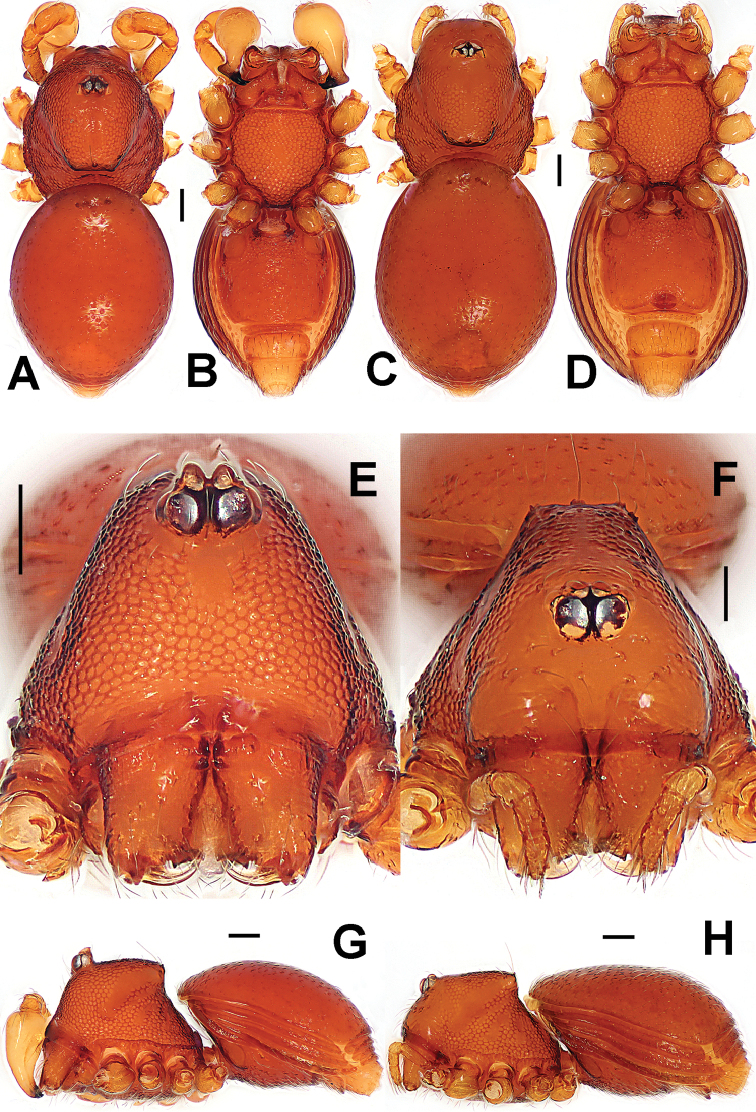
*Ablemma malacca* sp. n., male holotype (**A–B, E, G**) and female paratype (**C–D, F, H**). **A–D, G–H** habitus **E–F** prosoma. **A, C** dorsal **B, D** ventral **E–F** anterior **G–H** lateral. Scale bars: 0.10 mm.

**Figure 8.**
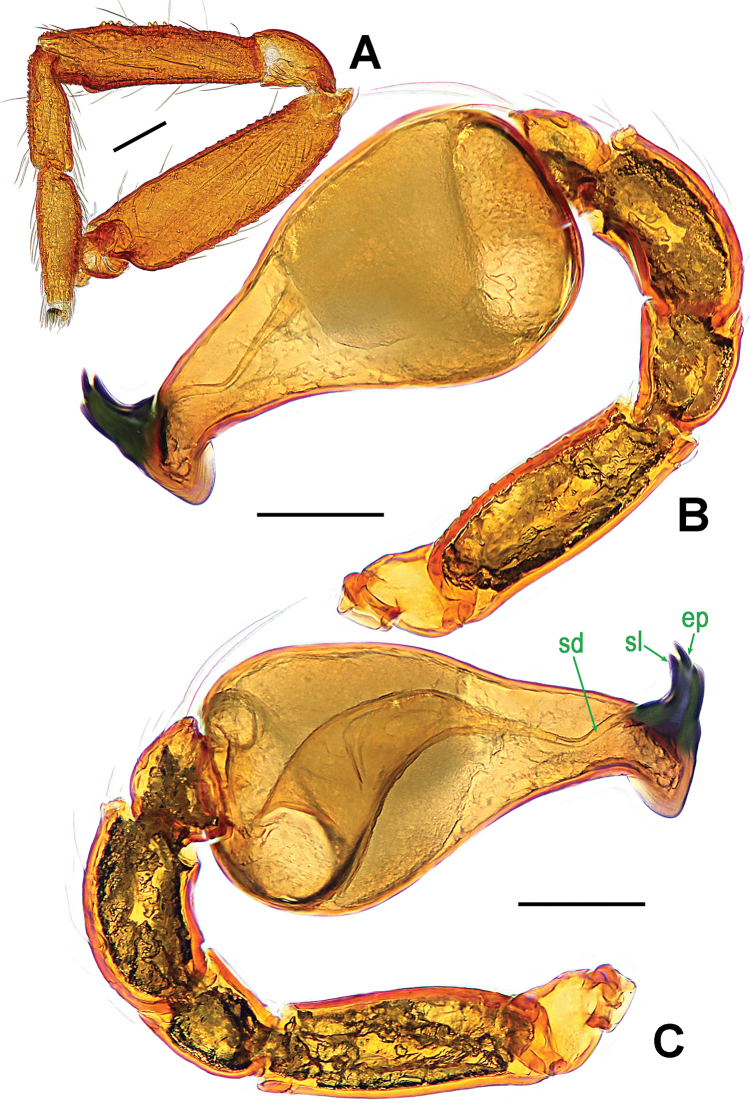
*Ablemma malacca* sp. n., male holotype. **A** left leg I, retrolateral **B** left palp, retrolateral **C** *ditto*, prolateral. Abbreviations: ep = embolic part of apes of palpal organ; sd = spermatic duct; sl = subterminal lamella. Scale bars: 0.10 mm.

**Figure 9.**
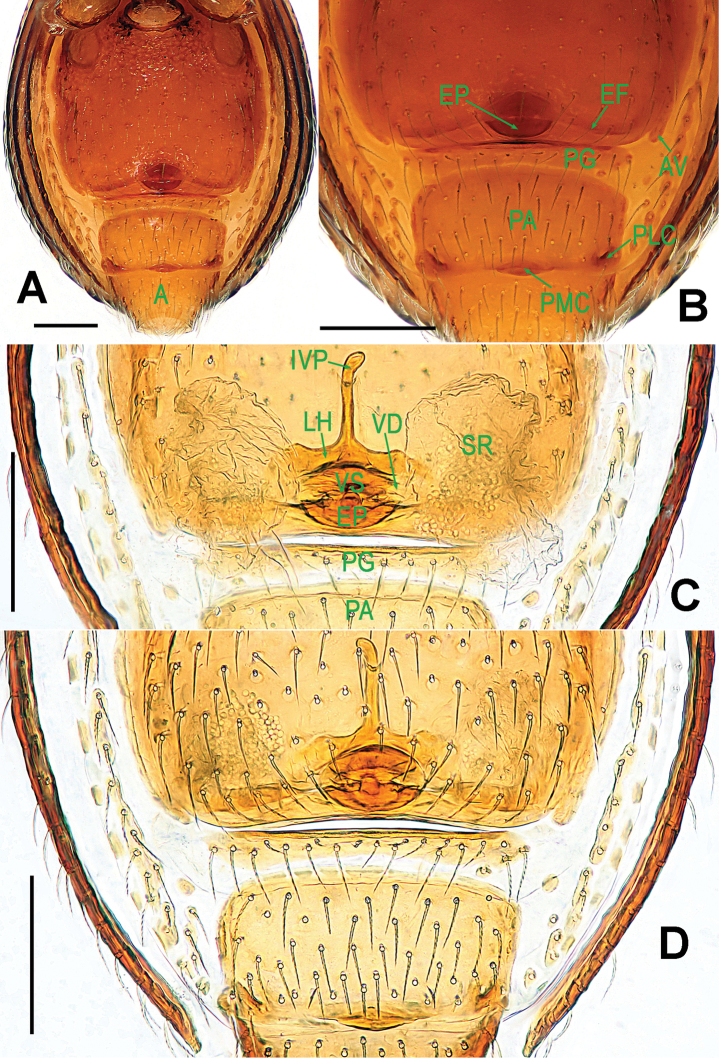
*Ablemma malacca* sp. n., female paratype. **A** opisthosoma **B** genital area (untreated) **C** cleared vulva (lactic acid-treated) **D** *ditto*. **A–B, D** ventral **C** dorsal. Abbreviations: A = anal plate; AV = anterior ventrolateral plate; EF = epigynal fold; EP = epigynal pit; IVP = inner vulval plate; LH = lateral horn; PA = preanal plate; PG = postgenital plate; PLC = posterolateral corner of PA; PMC = posteromedial corner of PA; SR = seminal receptaculum; VD = vulval duct; VS = vulval stem. Scale bars: 0.10 mm.

This new species can be distinguished from all its congeners with the exception of *Ablemma datahu* Lehtinen, 1981, *Ablemma makiling* Lehtinen, 1981, *Ablemma shimojanai* (Komatsu, 1968), *Ablemma singalang* Lehtinen, 1981, and *Ablemma unicornis* Burger, 2008 by the absence of a long, pointed tooth on male cephalic area posteriorly (Fig. 7E), the elongated palpal bulb (Fig. 8B–C), and by the long, claviform inner vulval plate (Fig. 9C). It differs from *Ablemma datahu* (see Lehtinen, 1981: 48, figs 176, 190, 196) and *Ablemma makiling* (see Lehtinen, 1981: 50, figs 192, 187) by the trifurcated embolic end (Fig. 8C) and the narrower, longer and straighter inner vulval plate (Fig. 9C); from *Ablemma shimojanai* (see Shear, 1978: 32, figs 82–87) by the lack of cheliceral horn and posteriorly cephalic process in male (Fig. 7E, G), the larger embolus (Fig. 8B–C), the inner vulval plate with a bent end and the stouter lateral horns (Fig. 9C); from *Ablemma singalang* (see Lehtinen, 1981: 48, fig. 170) by the shorter, trifurcated embolus (Fig. 8B–C); and from *Ablemma unicornis* (see Burger, 2008b: 253, figs 1, 2, 5, 11, 13) by the higher cephalic process in male ocular area (Fig. 7E), the six eyes in both sexes (Fig. 7A, C), the longer embolus (Fig. 8B–C) and the narrower base of inner vulval plate (Fig. 9C).

##### Description.

**Male** (holotype). Coloration: body brownish-yellow; legs yellowish-orange.

Measurements: total length 1.08; carapace 0.49 long, 0.44 wide, 0.47 high; abdomen 0.66 long, 0.50 wide, 0.40 high; clypeus 0.24 high; sternum 0.31 long, 0.30 wide. Length of legs: I 1.14 (0.39, 0.13, 0.28, 0.16, 0.18); II 1.01 (0.33, 0.12, 0.23, 0.16, 0.17); III 0.88 (0.28, 0.11, 0.18, 0.17, 0.14); IV 1.19 (0.37, 0.13, 0.30, 0.22, 0.17).

Prosoma (Fig. 7A–B, E, G): carapace finely reticulated, margin rugose; eyes white, ALE>PME>PLE, ocular area protruded (Fig. 7E, G); clypeus very high, sloping forward, marginally rounded; cephalic part raised, flat top; thoracic part radial furrow distinctly smooth; chelicerae robust, with a small basal tuber and an anterodistal tooth (Fig. 7E), cheliceral lamina well developed; labium triangular, distally blunt; sternum finely reticulated, with sparse setae. Legs: cuticle striated; femur I slightly swollen, tibia I with a distal-laterally ventral tuber (Fig. 8A).

Opisthosoma (Fig. 7A–B, G): dorsal scutum oval, dimpled with tiny pits, smooth between the pits, covered with sparse setae; ventral scutum rugose; perigenital scutum absent; postepigastral scutum straight, nearly same width as preanal scutum; preanal scutum rectangular, with thick posterolateral corners.

Palp (Fig. 8B–C): femoral cuticle sculptured and ventrally granulated, approximately 2 times longer than patella; tibia not swollen, with a distally dorsal trichobothrium; cymbium small, cup-shaped; bulb long pear-shaped, its surface smooth; spermatic duct basally broad, distally narrow; embolus short, foot-shaped, distally strongly sclerotized, and forming a trifurcated terminal.

**Female** (one of paratypes). Coloration: same as in male.

Measurements: total length 1.10; carapace 0.52 long, 0.42 wide, 0.42 high; abdomen 0.72 long, 0.54 wide, 0.42 high; clypeus 0.14 high; sternum 0.31 long, 0.31 wide. Length of legs: I 1.14 (0.38, 0.14, 0.25, 0.17, 0.20); II 1.00 (0.32, 0.13, 0.22, 0.16, 0.17); III 0.88 (0.26, 0.12, 0.17, 0.18, 0.15); IV 1.16 (0.36, 0.13, 0.29, 0.20, 0.18).

Prosoma (Fig. 7C–D, F, H): clypeus lower than in male, smooth, bears sparse setae; ocular area not protruded; cephalic part lower than in male, palps reduced; other features as in male.

Opisthosoma (Figs 7C–D, H; 9A–B): dorsal and ventral scuta as in male; lateral scutum I long, extending beyond posterior rim of booklung cover; cover centrally smooth, laterally rugose; postgenital scutum narrow, slightly curved; perigenital scutum absent; preanal scutum rectangular, with sparse serrated setae and one posteromedial and two posterolateral corners.

Genitalia (Fig. 9B–D): epigynal pit and vulval stem forming to an oval structure, strongly sclerotized (Fig. 9B, D); vulval duct and lateral horn weakly sclerotized, connected to the translucent, saccular seminal receptaculum (Fig. 9C); inner vulval plate long, distal end slightly bent (Fig. 9C); central process absent.

##### Distribution.

Singapore.

#### Genus Brignoliella

Taxon classificationAnimaliaAraneaeTetrablemmidae

Shear, 1978

##### Type species.

*Polyaspis acuminata* Simon, 1889 from New Caledonia (see Burger 2008a; Lehtinen 1981).

#### Brignoliella besutensis

Taxon classificationAnimaliaAraneaeTetrablemmidae

Lin, Li & Jäger, 2012

Figs 10 , 11 , 12

Brignoliella besutensis Lin et al., 2012: 56, figs 1A–F, 2A–C, 3A–C (male) from Malaysia.

##### Material examined.

1♂ and 8♀ (NHMSU), SINGAPORE: Pulau Ubin, altitude 2 m, 1°25'18.0"N, 103°56'25.4"E, 22 August 2015, S. Li and Y. Tong leg.

##### Diagnosis.

*Brignoliella besutensis* is similar to *Brignoliella caligiformis* Tong & Li, 2008 from Hainan Island, China (see Tong, 2013: 76, fig. 91A–G), but male can be distinguished by the non-inflated palpal tibia, the pear-shaped bulb, the horn-shaped embolus, and the slightly sinuous course of the spermatic duct (Fig. 11A–B, also see Lin et al., 2012: fig. 2A–C vs. Tong, 2013: fig. 91F–G). Female distinguished by the straight postgenital scutum (Fig. 12A–D vs. Tong, 2013: fig. 91D–E), the larger, adjacent pits of the preanal scutum (Fig. 12B–D vs. Tong, 2013: fig. 91D–E), the narrower lateral horn and the flatter vulval stem (Fig. 12C vs. Tong, 2013: fig. 91E).

##### Description.

**Male**. Coloration: body reddish-brown; legs pale reddish-brown to yellowish-brown.

Measurements: total length 1.16; carapace 0.58 long, 0.48 wide, 0.47 high; abdomen 0.72 long, 0.56 wide, 0.48 high; clypeus 0.16 high; sternum 0.32 long, 0.32 wide. Length of legs: I 1.25 (0.40, 0.14, 0.28, 0.21, 0.22); II 1.14 (0.36, 0.13, 0.25, 0.19, 0.21); III 1.01 (0.30, 0.12, 0.21, 0.19, 0.19); IV 1.29 (0.40, 0.13, 0.30, 0.26, 0.20).

**Figure 10.**
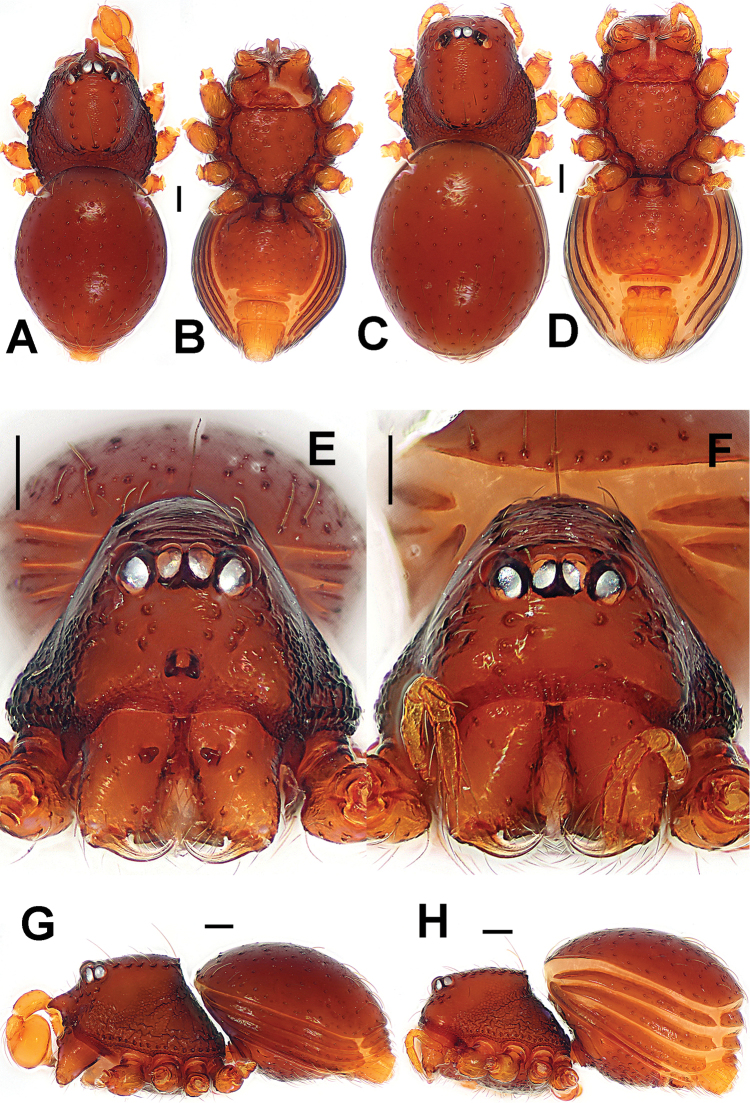
*Brignoliella besutensis* Lin, Li & Jäger, 2012, male (**A–B, E, G**) and female (**C–D, F, H**) from Singapore. **A–D, G–H** habitus **E–F** prosoma. **A, C** dorsal **B, D** ventral **E–F** anterior **G–H** lateral. Scale bars: 0.10 mm.

Prosoma (Fig. 10A–B, E, G): carapace strongly sclerotized; cephalic part smooth, slightly raised, with two rows of wart-like knots behind ocular area; thoracic part irregularly reticulated, margin rugose and denticulate; ocular area situated anteriorly, with six eyes in three diads, PME absent, ALE>AME=PLE in eye size. Clypeus with few wart-like knots, clypeal horn short, distally bifid; chelicerae surface smooth, with a fronto-mesial, short cheliceral apophysis, cheliceral lamina developed, translucent. Endites basally wide, distally narrow; labium distally blunt, subtriangular. Sternum long same as wide, with relatively large wart-like knots, marginally crinkled. Legs with lateral veins.

Opisthosoma (Fig. 10A–B, G): dorsal scutum oval, with sparse small pits, setae inserted in pits; ventral scutum anteriorly slightly crinkled, posteriorly with pits, booklung cover smooth; lateral scutum I long; postgenital scutum present, preanal scutum rectangular and smooth.

**Figure 11.**
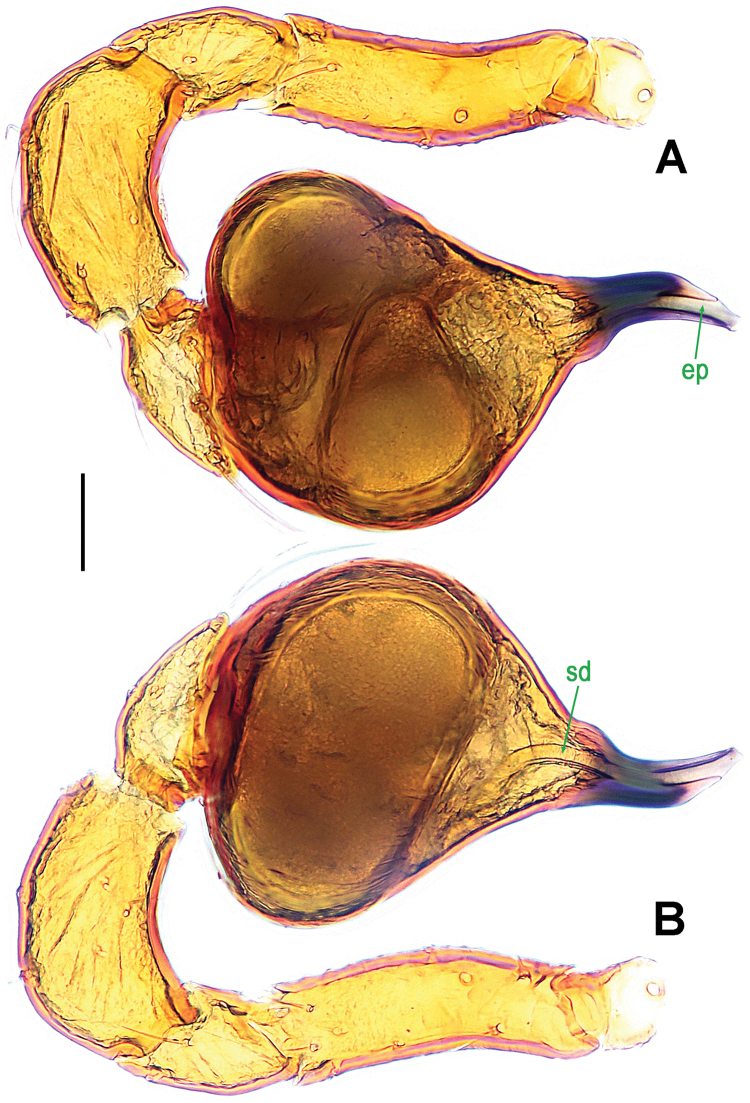
*Brignoliella besutensis* Lin, Li & Jäger, 2012, male from Singapore. **A** left palp, retrolateral **B** *ditto*, prolateral. Abbreviations: ep = embolic part of apes of palpal organ; sd = spermatic duct. Scale bars: 0.10 mm.

Palp (Fig. 11A–B): femur ventrally granulated, with three long setae; patella almost half as long as tibia, without modification; tibia slightly bent, with a distal-dorsal trichobothrium; cymbium subtriangular in lateral view; bulb pear-shaped; spermatic duct course simple; embolus short, sclerotized, distinctly narrowed and slightly bent distally, horn-shaped.

**Female** (First description). Coloration: same as in male.

Measurements: total length 1.18; carapace 0.56 long, 0.48 wide, 0.46 high; abdomen 0.84 long, 0.64 wide, 0.52 high; clypeus 0.12 high; sternum 0.33 long, 0.34 wide. Length of legs: I 1.22 (0.40, 0.14, 0.26, 0.20, 0.22); II 1.14 (0.36, 0.13, 0.24, 0.20, 0.21); III 0.98 (0.30, 0.12, 0.19, 0.20, 0.17); IV 1.26 (0.39, 0.13, 0.28, 0.24, 0.22).

Prosoma (Fig. 10C–D, F, H) as in male, but lacking clypeal horn and cheliceral apophysis, and clypeus lower than in male. Legs also as in male.

**Figure 12.**
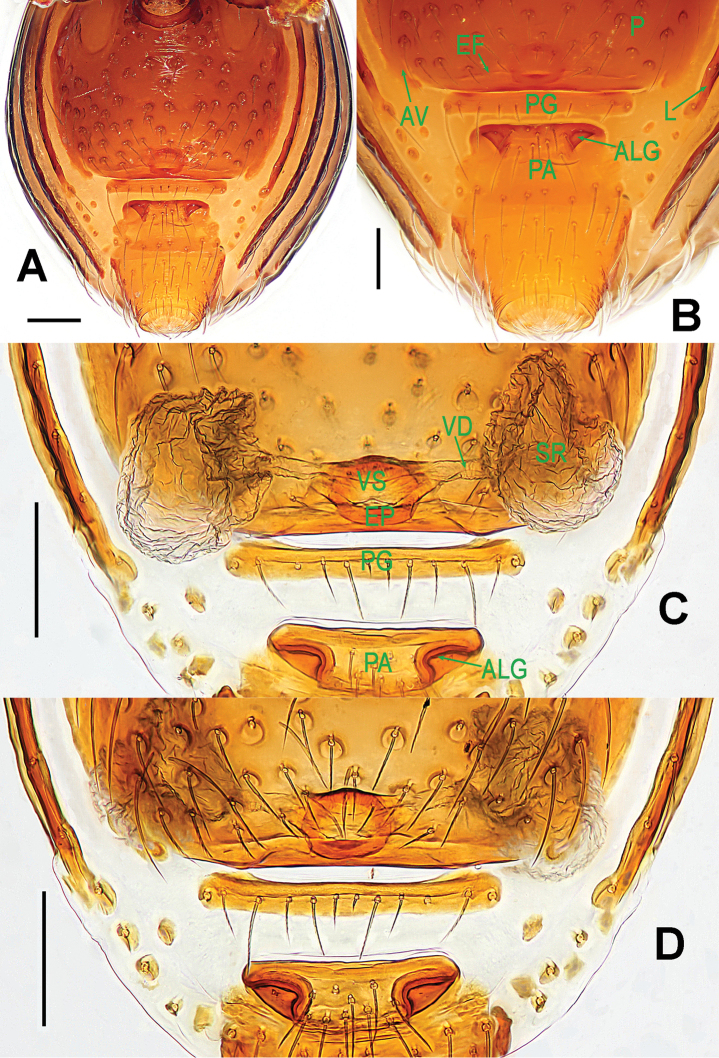
*Brignoliella besutensis* Lin, Li & Jäger, 2012, female from Singapore. **A** opisthosoma **B** genital area (untreated) **C** cleared vulva (lactic acid-treated) **D** *ditto*. **A–B, D** ventral **C** dorsal. Abbreviations: ALG = anterolateral groove of preanal plate; AV = anterior ventrolateral plate; EF = epigynal fold; EP = epigynal pit; L = lateral plate; P = pulmonary plate; PA = preanal plate; PG = postgenital plate; SR = seminal receptaculum; VD = vulval duct; VS = vulval stem. Scale bars: 0.10 mm.

Opisthosoma (Figs 10C–D, H; 12A–B): dorsal and ventral scuta as in male; lateral scutum I short, and not exceeding anterior margin of preanal scutum; postgenital scutum straight, slightly wider than preanal scutum; preanal scutum trapezoidal, with two large grooves at anterolateral corners (Fig. 12A–B).

Genitalia (Fig. 12C–D): epigynal fold distinct; epigynal pit and vulval stem forming into a sclerotized ring; central process and inner vulval plate absent; lateral horn and vulval duct weak, connected to the translucent, saccular seminal receptaculum (Fig. 12C).

##### Distribution.

Malaysia, Singapore.

#### Brignoliella michaeli

Taxon classificationAnimaliaAraneaeTetrablemmidae

Lehtinen, 1981

Figs 13 , 14 , 15

Brignoliella michaeli Lehtinen, 1981: 41, figs 103–106, 108, 111 (male and female) from Penang, Malaysia.

##### Material examined.

19♂ and 29♀ (NHMSU), SINGAPORE: Central Catchment Nature Reserve, near Mandai Agrotechnology Park, altitude 46 m, 1°24'53.7"N, 103°47'56.2"E, 1 September 2015, S. Li and Y. Tong leg.

##### Diagnosis.

**Figure 13.**
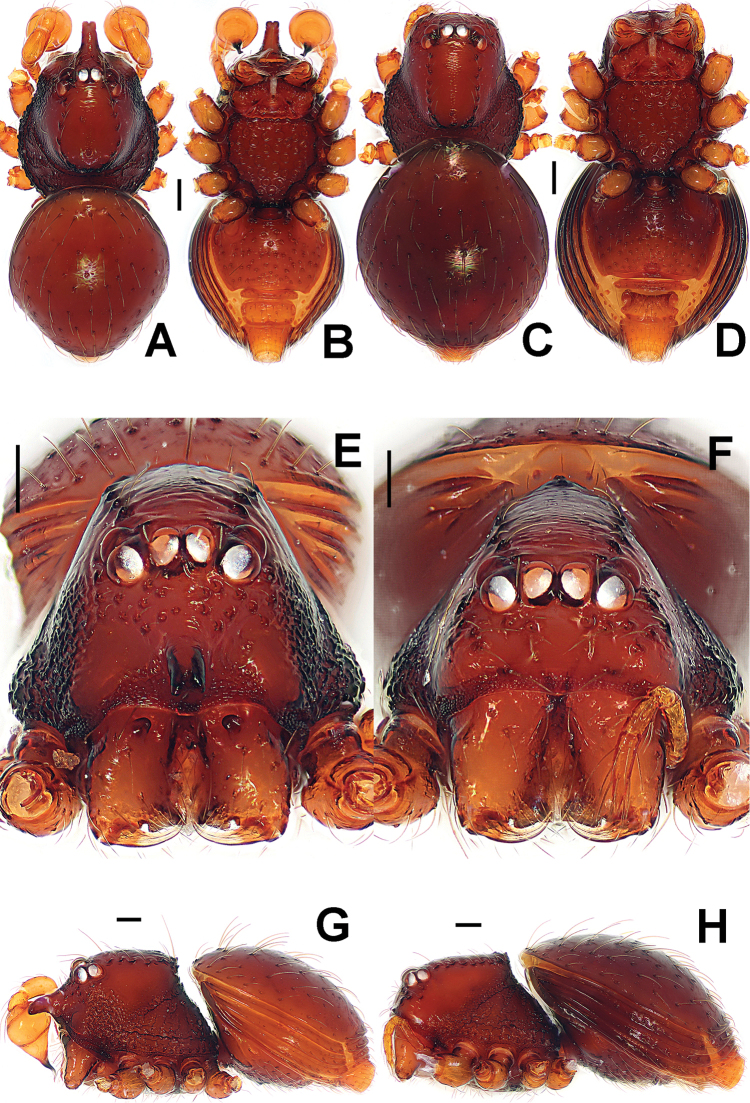
*Brignoliella michaeli* Lehtinen, 1981, male (**A–B, E, G**) and female (**C–D, F, H**) from Singapore. **A–D, G–H** habitus **E–F** prosoma. **A, C** dorsal **B, D** ventral **E–F** anterior **G–H** lateral. Scale bars: 0.10 mm.

**Figure 14.**
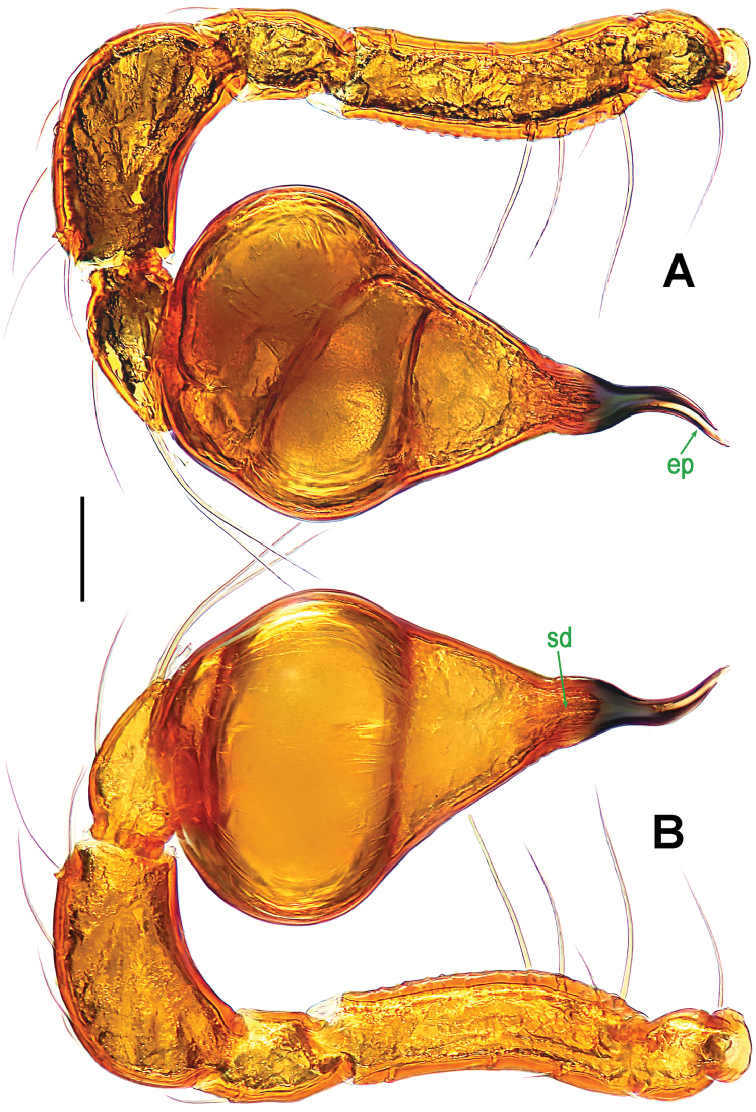
*Brignoliella michaeli* Lehtinen, 1981, male from Singapore. **A** left palp, retrolateral **B** *ditto*, prolateral. Abbreviations: ep = embolic part of apes of palpal organ; sd = spermatic duct. Scale bars: 0.10 mm.

**Figure 15.**
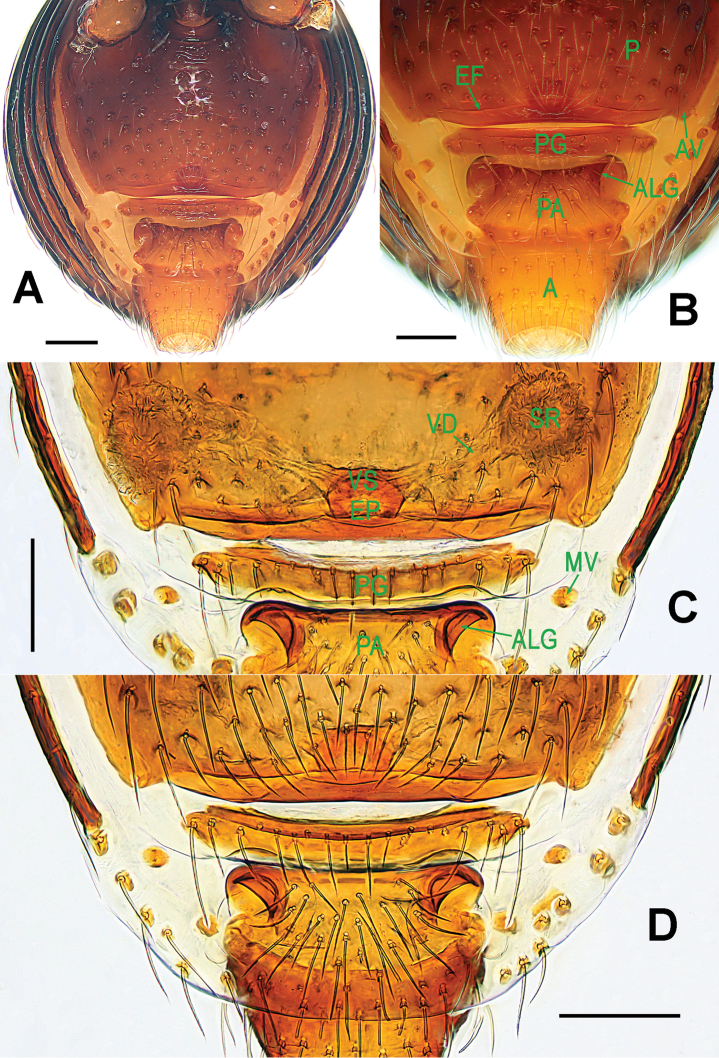
*Brignoliella michaeli* Lehtinen, 1981, female from Singapore. **A** opisthosoma **B** genital area (untreated) **C** cleared vulva (lactic acid-treated) **D** *ditto*. **A–B, D** ventral **C** dorsal. Abbreviations: A = anal plate; ALG = anterolateral groove of preanal plate; AV = anterior ventrolateral plate; EF = epigynal fold; EP = epigynal pit; MV = median ventrolateral plate; P = pulmonary plate; PA = preanal plate; PG = postgenital plate; SR = seminal receptaculum; VD = vulval duct; VS = vulval stem. Scale bars: 0.10 mm.

*Brignoliella michaeli* can be distinguished from all congeners with the exception of *Brignoliella besutensis*, *Brignoliella caligiformis*, *Brignoliella maoganensis* Tong & Li, 2008, *Brignoliella maros* Lehtinen, 1981, *Brignoliella martensi* (Brignoli, 1972), and *Brignoliella massai* Lehtinen, 1981 by the long, pear-shaped bulb or by the long, furcated clypeal horn. It differs from *Brignoliella besutensis* (Figs 10A–B, E; 11A–B, 12B–C), *Brignoliella caligiformis* (see Tong, 2013: 76, fig. 91A, E, F–G) and *Brignoliella maoganensis* (see Tong, 2013: 77, fig. 92A, E, F–G) by the longer clypeal horn in male, the more pointed embolus, the larger pits of preanal scutum, and the narrower lateral horn (Figs 13A–B, E; 14A–B, 15B–C); from *Brignoliella maros* (see Lehtinen, 1981: 39, figs 97, 116, 125) and *Brignoliella massai* (see Lehtinen, 1981: 40, figs 93, 95, 115, 124) by the longer clypeal horn and larger cheliceral horn in male (Fig. 13A–B, E), the longer pear-shaped bulb, the curving embolus (Fig. 14A–B) and the wider vulval stem (Fig. 15C); and from *Brignoliella martensi* (see Lehtinen, 1981: 38, figs 98, 112, 117) by the narrower, longer clypeal horn in male (Fig. 13A), the longer bulb and the curving, tapering embolus (Fig. 14A–B), and the nearly quadrate preanal scutum and the distinct epigynal fold (Fig. 15A–B, D).

##### Description.

**Male**. Coloration: body brownish-yellow; legs yellowish-orange.

Measurements: total length 1.38; carapace 0.74 long, 0.56 wide, 0.60 high; abdomen 0.82 long, 0.65 wide, 0.56 high; clypeus 0.20 high; sternum 0.37 long, 0.38 wide. Length of legs: I 1.45 (0.46, 0.16, 0.34, 0.25, 0.24); II 1.34 (0.42, 0.15, 0.30, 0.24, 0.23); III 1.21 (0.36, 0.14, 0.26, 0.23, 0.22); IV 1.53 (0.48, 0.15, 0.36, 0.30, 0.24).

Prosoma (Fig. 13A–B, E, G): carapace strongly sclerotized; cephalic part smooth, distinctly raised, with two rows of wart-like knots behind ocular area; thoracic cuticle irregularly rugose, marginally denticulate; ocular area situated anteriorly, with six eyes in three diads, PME absent, ALE>AME=PLE in eye size, ALE and PLE adjacent. Clypeus with wart-like knots, anteromargin rugose, clypeal horn long, distally bifid; chelicerae surface smooth, with a fronto-subbasal, short cheliceral apophysis, cheliceral lamina developed, translucent. Endites basally wide, distally narrow; labium subtriangular. Sternum long same as wide, with finely large ring-like pits, marginally crinkled. Legs with shallow annular grains.

Opisthosoma (Fig. 13A–B, G): dorsal scutum short oval, with sparse small pits, setae inserted in pits, smooth between the pits (Fig. 13A, E); ventral scutum modified by ring-like pits, booklung cover smooth; lateral scutum I long, and just over anteromargin of preanal scutum; postgenital scutum present, same wide as preanal scutum; preanal scutum rectangular, with an anterior long fold and two anterolaterally shallow grooves (Fig. 13B).

Palp (Fig. 14A–B): femur slightly bent, ventral cuticle granulated, approx. 2.2 times patella in length; tibia slightly swollen, approx. 2/3 times femur in long, with a distal-dorsal trichobothrium; cymbium short as patella; bulb pear-shaped, its surface with filamentous veins (Fig. 14A); spermatic duct base wide, and tapering to the apex of bulb after coiled a loop; embolus strongly sclerotized, mesially bent and distally sharp.

**Female**. Coloration: same as in male, but deeper in opisthosoma.

Measurements: total length 1.34; carapace 0.66 long, 0.56 wide, 0.56 high; abdomen 0.90 long, 0.72 wide, 0.66 high; clypeus 0.14 high; sternum 0.36 long, 0.38 wide. Length of legs: I 1.55 (0.50, 0.17, 0.35, 0.27, 0.26); II 1.44 (0.46, 0.16, 0.32, 0.26, 0.24); III 1.29 (0.39, 0.15, 0.28, 0.25, 0.22); IV 1.63 (0.51, 0.16, 0.38, 0.32, 0.26).

Prosoma (Fig. 13C–D, F, H) as in male; but lack of clypeal horn and cheliceral apophysis, and clypeus lower than in male. Legs also as in male.

Opisthosoma (Figs 13C–D, H; 15A–B): dorsal and ventral scuta as in male; lateral scutum I short, not extending beyond anteromargin of preanal scutum; postgenital scutum straight, wider than preanal scutum and in male, with an anterior fold; preanal scutum sub-rectangular, with two large, inverted, pocket-shaped grooves at anterolateral corners (Fig. 15A–B, D).

Genitalia (Fig. 15C–D): epigynal fold long; epigynal pit large, and vulval stem short; central process and inner vulval plate absent; lateral horn and vulval duct narrow, translucent, connected to the rugose, saccular seminal receptaculum (Fig. 15C).

##### Distribution.

Malaysia, Singapore.

#### Genus Singaporemma

Taxon classificationAnimaliaAraneaeTetrablemmidae

Shear, 1978

##### Type species.

*Singaporemma singulare* Shear, 1978 from Singapore (see Shear 1978).

#### Singaporemma lenachanae

Taxon classificationAnimaliaAraneaeTetrablemmidae

Lin & Li sp. n.

http://zoobank.org/0A6AB9AF-31B6-4153-A353-5AA16C45E72D

**Figure 16.**
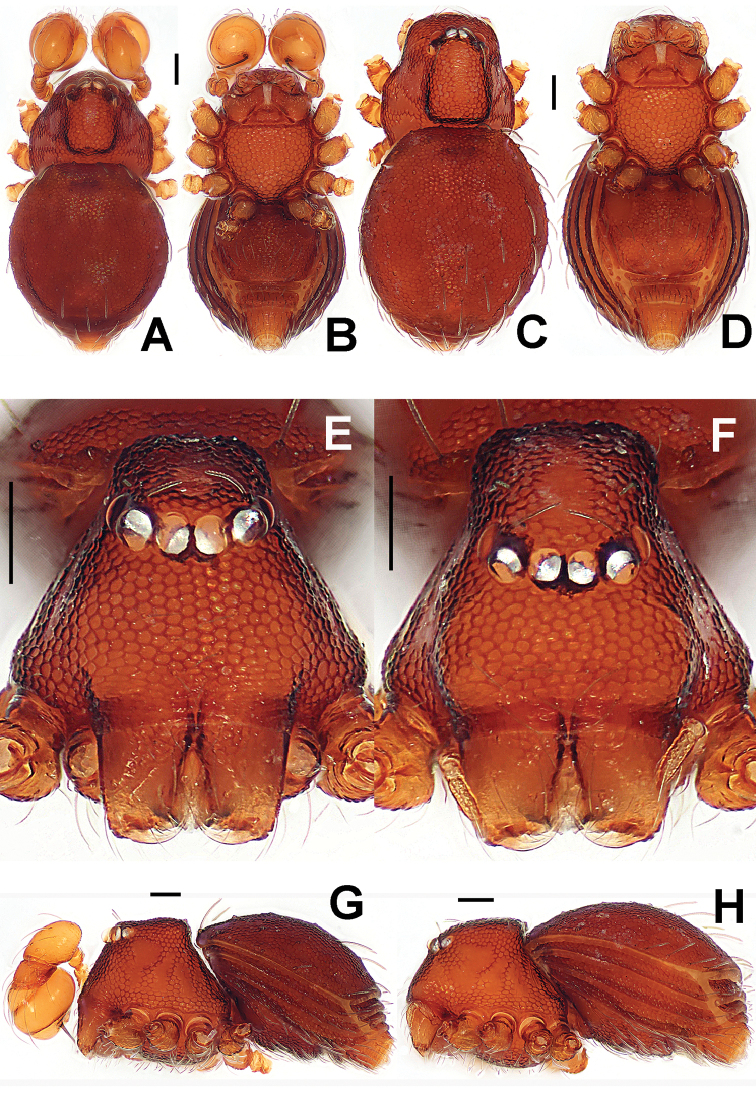
*Singaporemma lenachanae* sp. n., male holotype (**A–B, E, G**) and female paratype (**C–D, F, H**). **A–D, G–H** habitus **E–F** prosoma. **A, C** dorsal **B, D** ventral **E–F** anterior **G–H** lateral. Scale bars: 0.10 mm.

**Figure 17.**
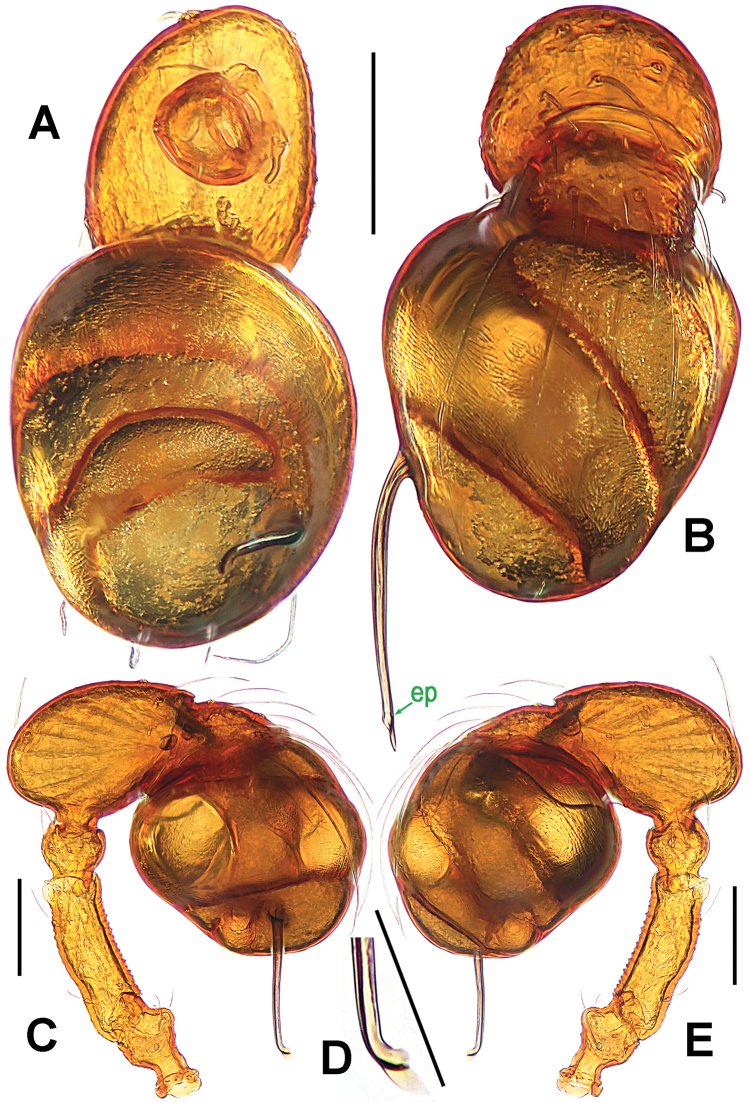
*Singaporemma lenachanae* sp. n., male holotype. **A** palpal bulb, ventral **B** left palp, anterior **C** *ditto*, prolateral **D** embolic end, prolateral **E** left palp, retrolateral. Abbreviation: ep = embolic part of apes of palpal organ. Scale bars: 0.10 mm.

**Figure 18.**
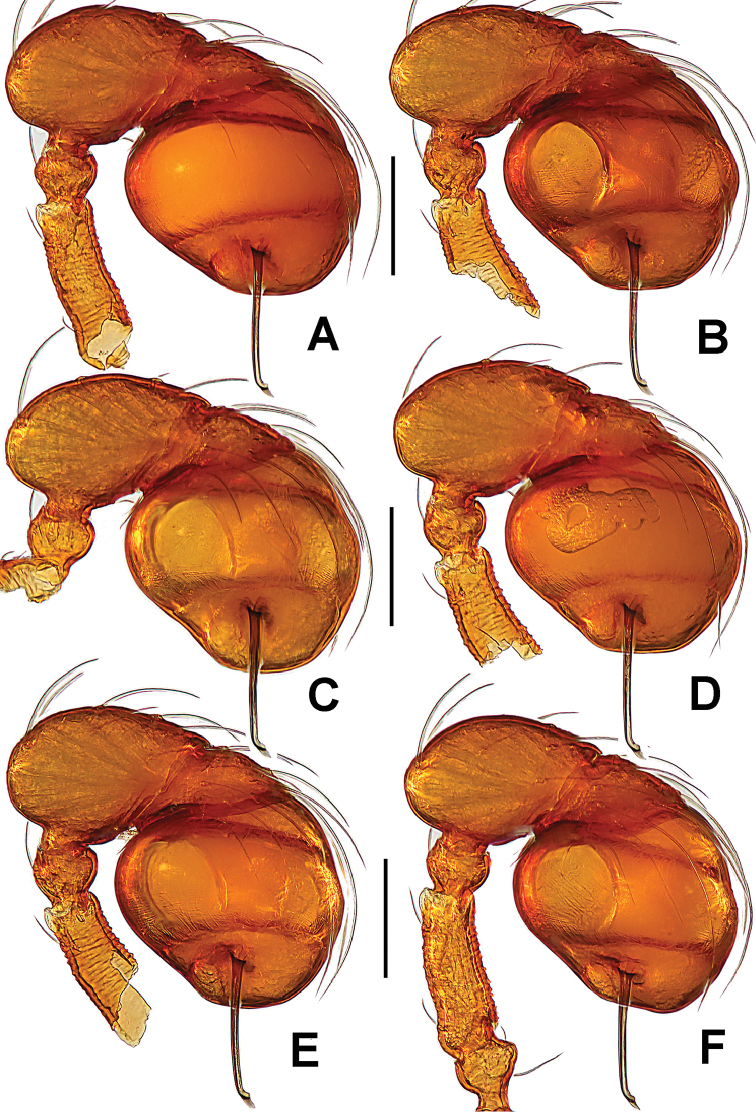
*Singaporemma lenachanae* sp. n., male paratypes. **A–F** left palps, prolateral. Scale bars: 0.10 mm.

**Figure 19.**
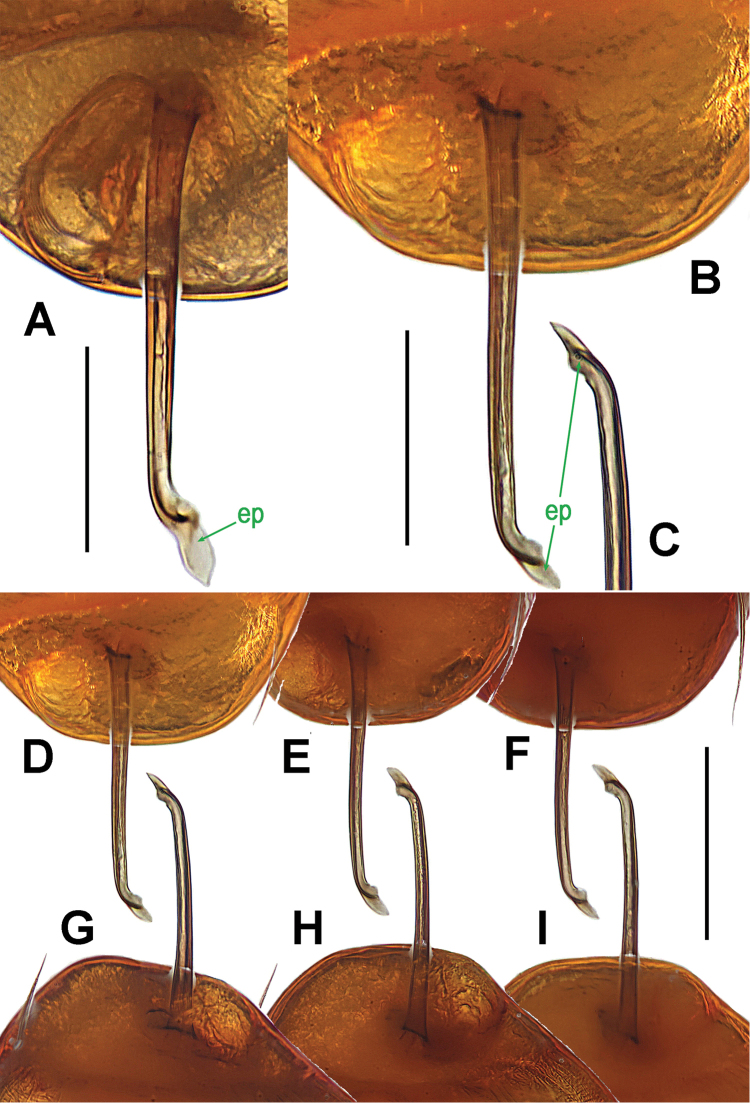
*Singaporemma halongense* Lehtinen, 1981, male paratype (**A**), and *Singaporemma lenachanae* sp. n., male paratypes (**B–I**). **A** embolic end, prolateral **B–I** *ditto*, prolateral. Abbreviations: ep = embolic part of apes of palpal organ. Scale bars: 0.10 mm.

**Figure 20.**
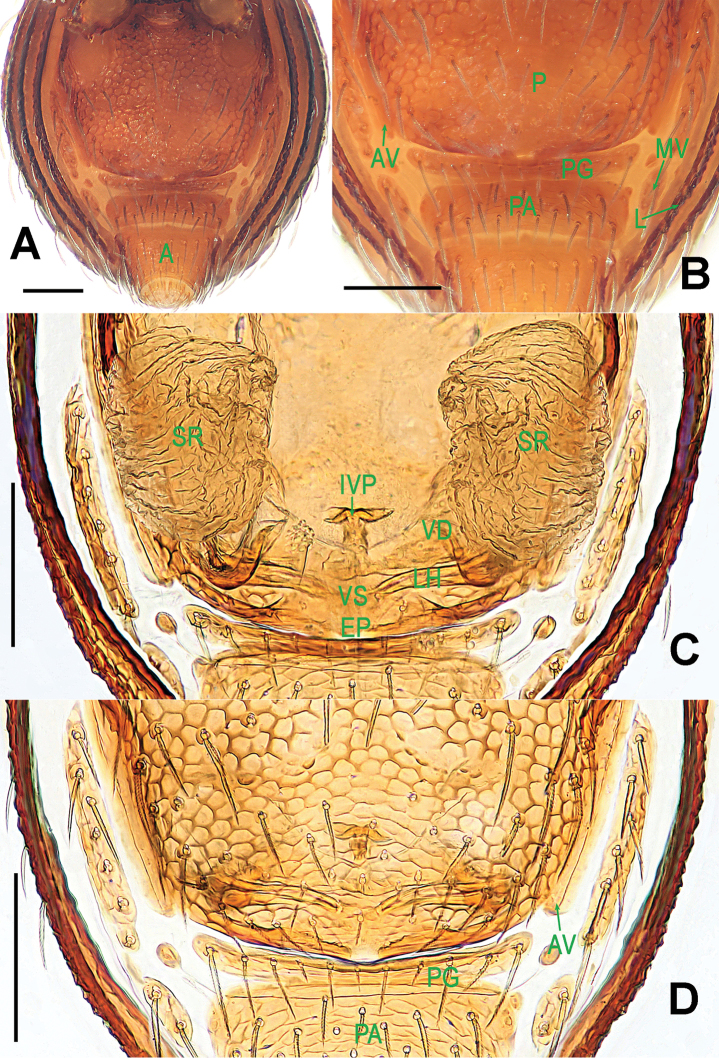
*Singaporemma lenachanae* sp. n., female paratype. **A** opisthosoma **B** genital area (untreated) **C** cleared vulva (lactic acid-treated) **D** *ditto*. **A–B, D** ventral **C** dorsal. Abbreviations: A = anal plate; AV = anterior ventrolateral plate; EP = epigynal pit; IVP = inner vulval plate; L = lateral plate; LH = lateral horn; MV = median ventrolateral plate; PA = preanal plate; PG = postgenital plate; SR = seminal receptaculum; VD = vulval duct; VS = vulval stem. Scale bars: 0.10 mm.

Figs 16 , 17 , 18 , 19B–I , 20

##### Material.

**Holotype** ♂ (LKCNHM), SINGAPORE: Bukit Timah Nature Reserve, Seraya Loop, altitude 118 m, 1°21'25.4"N, 103°46'25.3"E, 17 August 2015, S. Li and Y. Tong leg. **Paratypes** 4♂ and 2♀ (LKCNHM), same data as holotype.

##### Other material examined.

3♂ and 2♀ (NHMSU), SINGAPORE: Bukit Timah Nature Reserve, Seraya Loop, altitude 118 m, N1°21'25.4", E103°46'25.3", 17 August 2015, S. Li and Y. Tong leg.

##### Other species studied for comparison.

**Figure 21.**
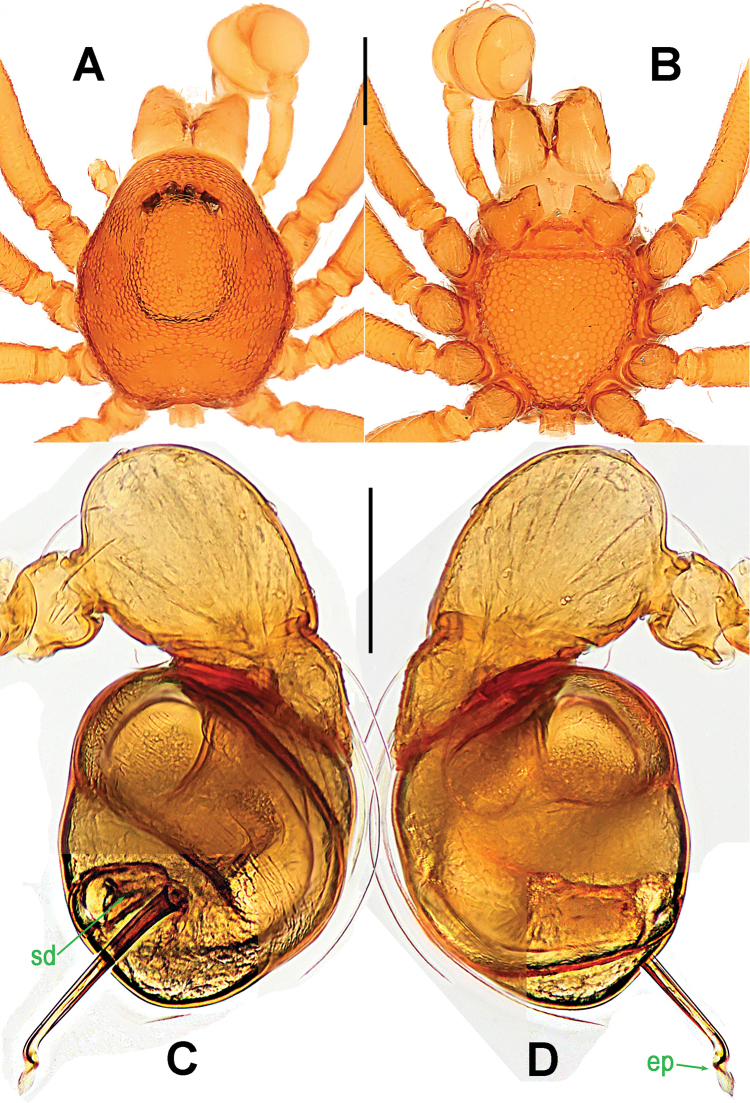
*Singaporemma halongense* Lehtinen, 1981, male paratype. **A** prosoma, dorsal **B** *ditto*, ventral **C** left palp, prolateral **D** *ditto*, retrolateral. Abbreviations: ep = embolic part of apes of palpal organ; sd = spermatic duct. Scale bars: 0.10 mm.

*Singaporemma halongense* Lehtinen, 1981 (Figs 19A, 21A–D; Lehtinen, 1981: 31, figs 43, 49, 54, 58, 62). Paratype 1♂ (ZMUT), VIETNAM: Quang Ninh, Ha Long, in litter of brook valley, altitude 30 m, 11 October 1978, P.T. Lehtinen leg.

##### Etymology.

Patronymic in honour of Dr Lena Chan from the National Biodiversity Centre, Singapore in recognition of her support of this study; noun (name) in genitive case.

##### Diagnosis.

This new species can be distinguished from all other congeners with the exception of *Singaporemma banxiaoensis* Lin & Li, 2014, *Singaporemma halongense* and *Singaporemma singulare* by the slender, straight embolus without any furcated end (Fig. 17B–E), the lack of central process, and the “T”-shaped inner vulval plate (Fig. 20C). It differs from *Singaporemma banxiaoensis* (see Lin and Li, 2014: 42, fig. 5A–D) by the lower initial position of embolus (Fig. 17A vs. fig. 5A), the wider embolic end (Fig. 17D, 19B–C vs. Fig. 5D), the wider, shorter preanal scutum (Fig. 20B vs. Fig. 6B), the narrower lateral horns, and the “T”-shaped inner vulval plate (Fig. 20C vs. Fig. 6C). Differs from *Singaporemma halongense* (see Figs 19A, 21A–D, and Lehtinen, 1981: 31, fig. 58) by the sharper, narrower embolic end (Fig. 19B–I vs. Fig. 19A), the wider preanal scutum and the “T”-shaped inner vulval plate (Fig. 20B–C vs. fig. 58). From *Singaporemma singulare* (see Figs 23A–E, 25A–E) by the shorter ovate bulb (Fig. 18A–F vs. Fig. 23A–C, 25D–E), the bent embolic end (Fig. 17D, 19B–I vs. Fig. 23D, 25C), the more swollen palpal tibia (Fig. 18A–F vs. Fig. 23A–B, 25D–E), the narrower postgenital scutum and preanal scutum (Fig. 20B vs. Fig. 23H, 26B), and the larger inner vulval plate (Fig. 20C vs. Fig. 26C).

##### Description.

**Male** (holotype). Coloration: body reddish-brown; legs yellowish-brown.

Measurements: total length 0.86; carapace 0.42 long, 0.38 wide, 0.40 high; abdomen 0.58 long, 0.49 wide, 0.39 high; clypeus 0.17 high; sternum 0.24 long, 0.27 wide. Length of legs: I 0.95 (0.31, 0.11, 0.22, 0.15, 0.16); II 0.87 (0.27, 0.10, 0.20, 0.15, 0.15); III 0.77 (0.24, 0.10, 0.16, 0.14, 0.13); IV 0.99 (0.33, 0.11, 0.23, 0.17, 0.15).

Prosoma (Fig. 16A–B, E, G): carapace finely reticulated; eyes white, AME=ALE>PLE in size, ARE procured; clypeus high, sloping forward, marginally rounded (Fig. 16C, 16G); cephalic part raised, quadrate (Fig. 16A); thoracic part smooth in radial grooves; Chelicerae without any horn or process, cheliceral lamina developed; endites basally wide, distally narrow; labium triangular, distally blunt; sternum finely reticulated, scutellate, posterior corner blunt, margin rugose, with sparse setae. Legs cuticle striated.

Opisthosoma (Fig. 16A–B, G): covered with serrated setae; dorsal scutum oval, but anteriorly truncated, finely reticulated; ventral scutum reticulated; lateral scutum long, and exceeding beyond the posterior margin of preanal scutum; perigenital scutum present; postgenital scutum straight, narrow, same wide as preanal scutum; preanal scutum shallow rectangular.

Palp (Figs 17A–E, 18A–F, 19B–I): femoral cuticle sculptured and granulated, approximately 2.5 times patella in length; patella short, and small; tibia remarkably swollen, about 2.3 times femur in width (Fig. 17B–C, E); cymbium relatively small, bearing long setae; bulb egg-shaped, its surface with irregular lines (Fig. 17A–B); embolus long, straight, starting at the subapical 1/3 position of bulb prolaterally (Fig. 17B–C, 18A–F), embolic end slightly bent, sharp and flexible (Figs 17D, 19B–I).

**Female** (one of paratypes). Coloration: same as in male.

Measurements: total length 0.96; carapace 0.44 long, 0.38 wide, 0.41 high; abdomen 0.64 long, 0.52 wide, 0.40 high; clypeus 0.15 high; sternum 0.26 long, 0.27 wide. Length of legs: I 0.91 (0.28, 0.12, 0.20, 0.15, 0.16); II 0.83 (0.26, 0.11, 0.18, 0.14, 0.14); III 0.76 (0.24, 0.10, 0.16, 0.13, 0.13); IV 0.98 (0.32, 0.11, 0.22, 0.17, 0.16).

Prosoma (Fig. 16C–D, F, H) as in male, but clypeus slightly lower than in male. Palps distinctly reduced. Legs also as in male.

Opisthosoma (Figs 16C–D, H; 20A): dorsal and ventral scuta as in male; lateral scutum I long, extending beyond posterior margin of preanal scutum; perigenital scuta small, oval; postgenital scutum slightly curved, wider than preanal scutum; preanal scutum sculptured, shallow rectangular, with sparse serrated setae.

Genitalia (Fig. 20B–D): epigynal pit distinct, oval (Fig. 20B, D); vulval posterior margin weakly sclerotized (Fig. 20C); vulval stem triangular; central process absent; inner vulval plate “T”-shaped (Fig. 20C); lateral horn narrow, and weakly sclerotized; vulval ducts relatively wide, membranous and translucent, connected to the rugose, large, saccular seminal receptaculum (Fig. 20C).

##### Distribution.

Singapore.

#### Singaporemma singulare

Taxon classificationAnimaliaAraneaeTetrablemmidae

Shear, 1978

**Figure 22.**
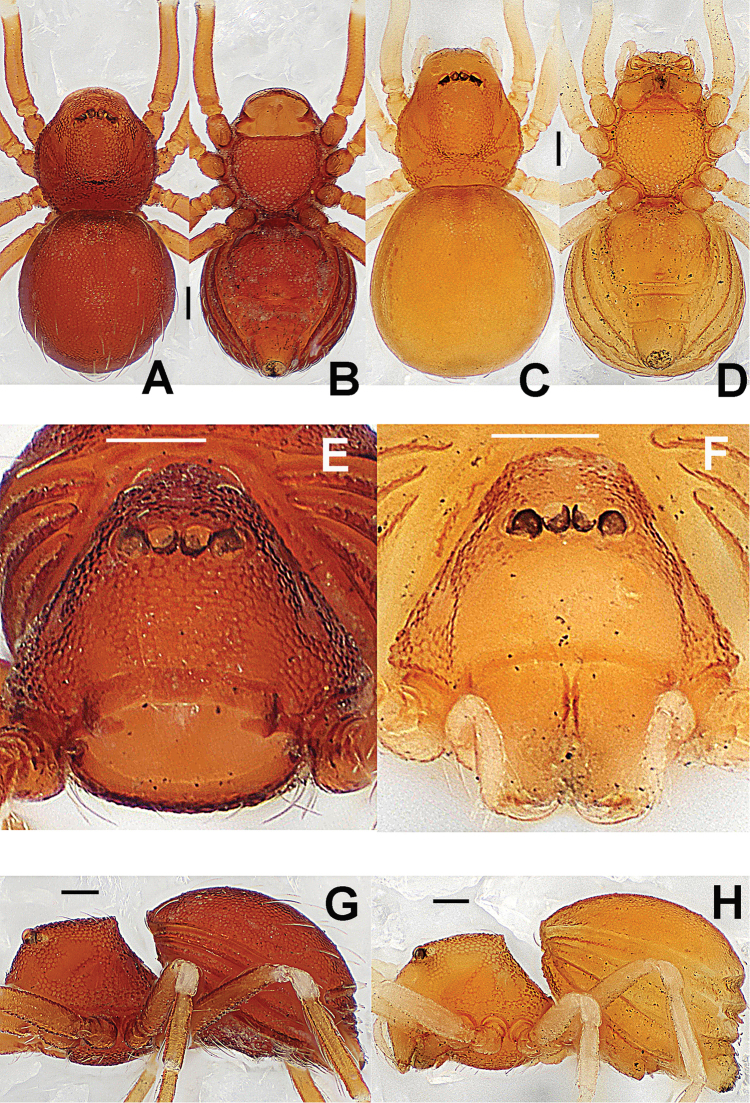
*Singaporemma singulare* Shear, 1978, male holotype (**A–B, E, G**) and female paratype (**C–D, F, H**). **A–D, G–H** habitus **E–F** prosoma. **A, C** dorsal **B, D** ventral **E–F** anterior **G–H** lateral. Scale bars: 0.10 mm.

**Figure 23.**
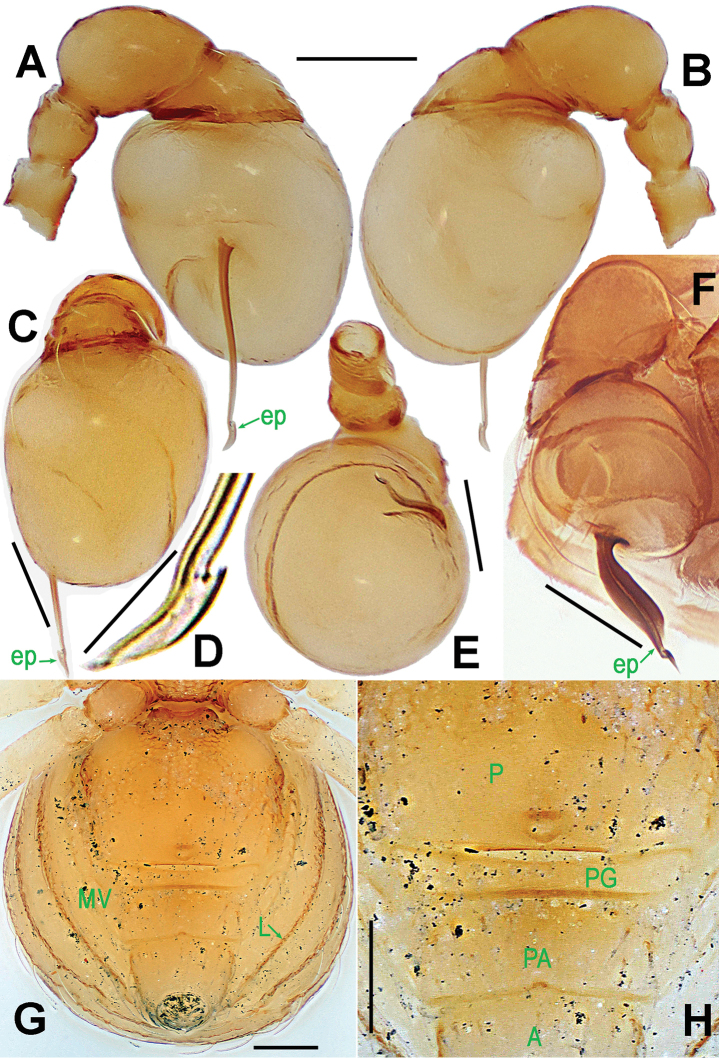
*Singaporemma singulare* Shear, 1978, male holotype (**A–E)** and female paratype **(G–H**), and *Singaporemma adjacens* Lehtinen, 1981, male holotype (**F**). **A–C, E–F** left palp **D** embolic end **G** opisthosoma **H** Genital area (untreated). **A, D** prolateral **B, F** retrolateral **C** anterior **E, G–H** ventral. Abbreviations: A = anal plate; ep = embolic part of apes of palpal organ; L = lateral plate; MV = median ventrolateral plate; P = pulmonary plate; PA = preanal plate; PG = postgenital plate. Scale bars: 0.10 mm, **D** 0.05 mm .

**Figure 24.**
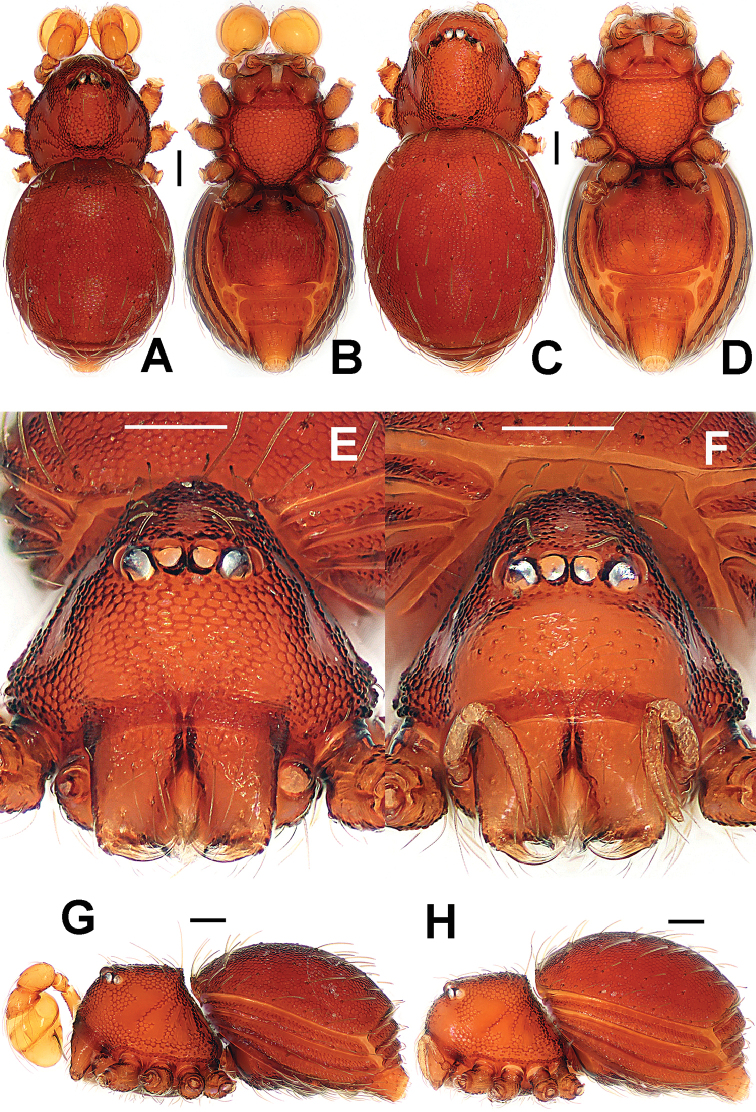
*Singaporemma singulare* Shear, 1978, male and female specimens from Singapore. **A–D, G–H** habitus **E–F** prosoma. **A, C** dorsal **B, D** ventral **E–F** anterior **G–H** lateral. Scale bars: 0.10 mm.

**Figure 25.**
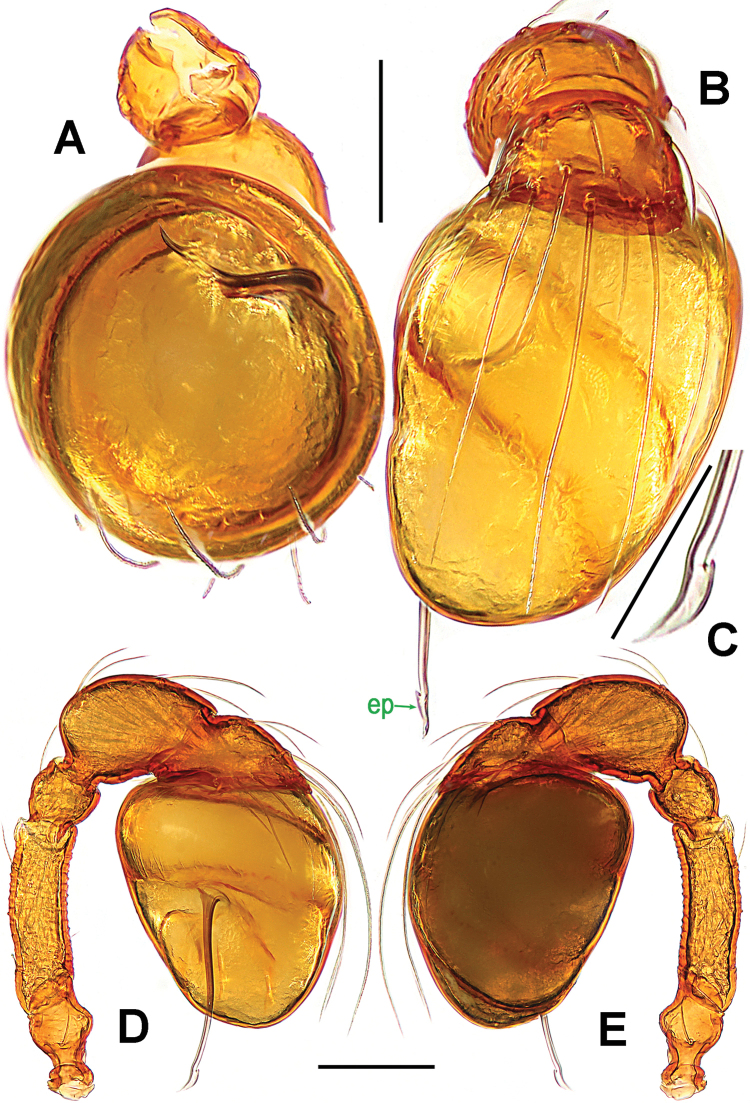
*Singaporemma singulare* Shear, 1978, male specimen from Singapore. **A** palpal bulb, ventral **B** left palp, anterior **C** embolic end, prolateral **D** left palp, prolateral **E** *ditto*, retrolateral. Abbreviations: ep = embolic part of apes of palpal organ. Scale bars: 0.10 mm.

**Figure 26.**
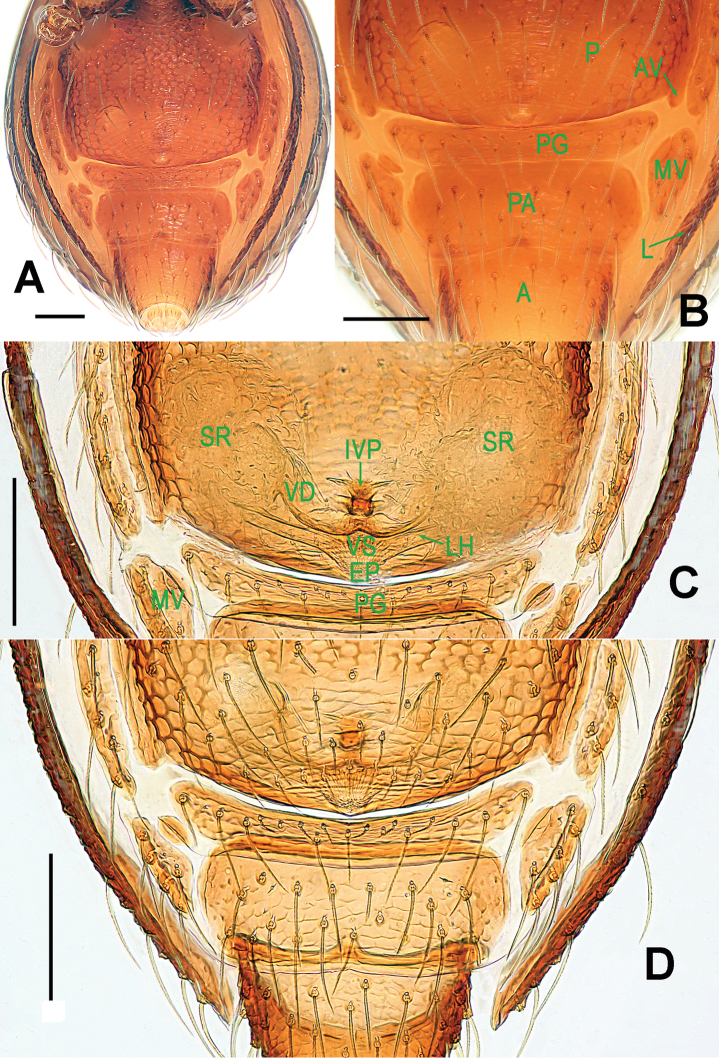
*Singaporemma singulare* Shear, 1978 , female specimen from Singapore. **A** opisthosoma **B** genital area (untreated) **C** cleared vulva (lactic acid-treated) **D** *ditto*. **A–B, D** ventral **C** dorsal. Abbreviations: A = anal plate; AV = anterior ventrolateral plate; EP = epigynal pit; IVP = inner vulval plate; L = lateral plate; LH = lateral horn; MV = median ventrolateral plate; P = pulmonary plate; PA = preanal plate; PG = postgenital plate; SR = seminal receptaculum; VD = vulval duct; VS = vulval stem. Scale bars: 0.10 mm.

Figs 22 , 23A–E, G–H , 24 , 25 , 26

Singaporemma singularis Shear, 1978: 36, figs 108–111; Lehtinen, 1981: 31.

##### Examined materiral.

**Holotype** ♂ and **paratype** 1♀ (AMNH), SINGAPORE: near MacRitchie Reservoir, 25 October 1950, M.W.F. Tweedie leg.

##### Other material.

10♂ and 6♀ (LKCNHM), SINGAPORE: Central Catchment Nature Reserve, treetop walk, 1°21'13.3"N, 103°48'29.4"E, 28 August 2015, S. Li and Y. Tong leg; 5♂ and 4♀ (NHMSU), SINGAPORE: Central Catchment Nature Reserve, Treetop Walk, 1°21'13.3"N, 103°48'29.4"E , 28 August 2015, S. Li and Y. Tong leg.

##### Other species studied for comparison.

*Singaporemma adjacens* Lehtinen, 1981 (Fig. 23F; Lehtinen, 1981: 31, figs 47, 51, 64). Holotype ♂ (ZMUT), VIETNAM: Quang Ninh, Ha Long, in litter of dense jungle close to seashore, altitude 10 m, 12 October 1978, P.T. Lehtinen leg.

##### Diagnosis.

*Singaporemma singulare* is distinguished from *Singaporemma bifurcata* (see Lin and Li, 2010: 26, figs 35–37) and *Singaporemma wulongensis* (see Lin and Li, 2014: 46, fig. 8A–F) by the embolus without any furcate end; from *Singaporemma adjacens* (see Fig. 23F and Lehtinen, 1981: 31: fig. 64a–b) by the narrower embolus. It is similar to *Singaporemma banxiaoensis* (see Lin and Li 2014: 42, fig. 5A–D), *Singaporemma halongense* (see Figs 19A, 21C–D and Lehtinen, 1981: 31, fig. 62a–b), and *Singaporemma lenachanae* sp. n. (Figs 17A–E, 18A–F, 19B–I) in having a straight embolus with modified end, but the male can be distinguished by the initial position of embolus (Figs 23A, 25D) and the knife-shaped embolic end (Figs 23D, 25C). Female distinguished by the absence of central process (Fig. 26C) and the punctured rather than reticulated clypeal area (Fig. 24F).

##### Description.

**Male**. Coloration: body reddish-brown; legs yellowish-brown.

Measurements: total length 1.14; carapace 0.50 long, 0.45 wide, 0.44 high; abdomen 0.73 long, 0.60 wide, 0.52 high; clypeus 0.19 high; sternum 0.30 long, 0.33 wide. Length of legs: I 1.24 (0.40, 0.13, 0.30, 0.19, 0.22); II 1.10 (0.34, 0.12, 0.26, 0.18, 0.20); III 0.99 (0.30, 0.12, 0.22, 0.17, 0.18); IV 1.36 (0.43, 0.14, 0.34, 0.23, 0.22).

Prosoma (Fig. 24A–B, E, G): carapace finely reticulated, except for the radial grooves in thoracic area, marginally denticulate (Fig. 24A, E); eyes with black base, ALE>AME>PLE, ALE and PLE adjacent, ARE straight; cephalic part raised, top flat, covered with long setae (Fig. 24G); clypeus high, sharply sloping forward, bears densely short setae, anterior margin rugose (Fig. 24E); Cheliceral frontal surface sculptured, but lack of process, cheliceral lamina developed; sternum finely reticulated, marginally rugose, and posteriorly truncated. Legs striated, cuticle scalelike.

Opisthosoma (Figs 24A–B, G; 26A): covered with serrated setae; dorsal scutum oval, reticulated, bears sparse nodules and setae; ventral scutum reticulated, margin rugose; booklung cover rounded, smooth; lateral scutum I long, exceeding by far the posterior margin of preanal scutum; perigenital scutum large; postgenital scutum wide, its posterior margin overlaps joint of anterior margin of preanal scutum; preanal scutum rectangular, with blunt corners.

Palp (Figs 23A–E; 25A–E): femoral cuticle granular, striated, approximately 2.6 times as long as patella; patella short; tibia swollen, 1.8 times as wide as femur; cymbium wide and compressed; bulb long egg-shaped, surface smooth; spermatic duct basally wide, and tapering to the base of embolus after coiling a loop; embolus thin and long, weakly sclerotized, starting from the prolateral pericenter of bulbous surface, and almost straight downwards (Figs 23A, 25D); embolic end flexuous, knife-shaped (Figs 23D, 25C).

**Female**. Coloration: body slightly lighter than in male; legs yellowish-brown.

Measurement: total length 1.18; carapace 0.50 long, 0.44 wide, 0.44 high; abdomen 0.74 long, 0.63 wide, 0.56 high; clypeus 0.17 high; sternum 0.29 long, 0.32 wide. Length of legs: I 1.24 (0.40, 0.14, 0.28, 0.20, 0.22); II 1.14 (0.37, 0.13, 0.25, 0.19, 0.20); III 1.04 (0.32, 0.12, 0.22, 0.18, 0.20); IV 1.39 (0.44, 0.13, 0.35, 0.24, 0.23).

Prosoma (Fig. 24C–D, F, H) as in male, except for clypeal area no reticulated, but covered with short setae. Palp reduced. Legs also as in male.

Opisthosoma (Figs 24C–D, H; 26A): dorsal and ventral scuta as in male; lateral scutum I long, exceeding by far posterior margin of preanal scutum; perigenital scutum large, long oval; postgenital scutum slightly curved, faintly wider than preanal scutum, overlapped joint the anterior margin of preanal scutum; preanal scutum smooth, nearly rectangular, with sparse serrated setae.

Genitalia (Figs 23H, 26B–D): epigynal pit small, indistinct (Fig. 26B, D), closed to vulval posterior margin (Fig. 21C); vulval stem wide, connected with lateral horns; central process absent; inner vulval plate “T”-shaped, basally sclerotized (Fig. 26C); lateral horn narrow, and straight; vulval ducts relatively wide, upward curved, translucent, connected to the saccular seminal receptaculum (Fig. 26C).

##### Distribution.

Singapore.

##### Remarks.

This species is originally described from Singapore and was designated as the type species of the genus *Singaporemma* by Shear (1978). Based on the only male specimen available at that time, he had illustrated an embolus of the right palp that was said to be “curving sharply posteriorly” (Shear, 1978: 36, figs 109–110), i.e. bent at right angles at about mid-length. Schwendinger and Košulič (2015) suggested that Shear’s description of *Singaporemma singulare* was based on an atypical specimen. They noted that five other *Singaporemma* male specimens from Singapore, one of them from the type locality itself, all had “essentially straight emboli (only slightly bent ventrad) on both palps”. We have since reexamined the holotype of the species and photographed its left palp (Fig. 23A–E). We can now confirm that Shear (1978) had indeed described a deformed embolus.

#### Genus Sulaimania

Taxon classificationAnimaliaAraneaeTetrablemmidae

Lehtinen, 1981

##### Type species.

*Sulaimania vigelandi* Lehtinen, 1981 from Malaysia (see Lehtinen 1981).

#### Sulaimania brevis

Taxon classificationAnimaliaAraneaeTetrablemmidae

Lin & Li sp. n.

http://zoobank.org/B62F8B41-FE14-49A7-B5E3-DC75AD24A40C

Fig. 27

##### Material.

**Holotype** ♂ (LKCNHM), SINGAPORE: Bukit Timah Nature Reserve, Jungle Fall Stream, altitude 118 m, 1°21'25.4"N, 103°46'25.3"E, 21 August 2015, S. Li and Y. Tong leg. **Paratype** 1♂ (LKCNHM), same data as holotype.

**Figure 27.**
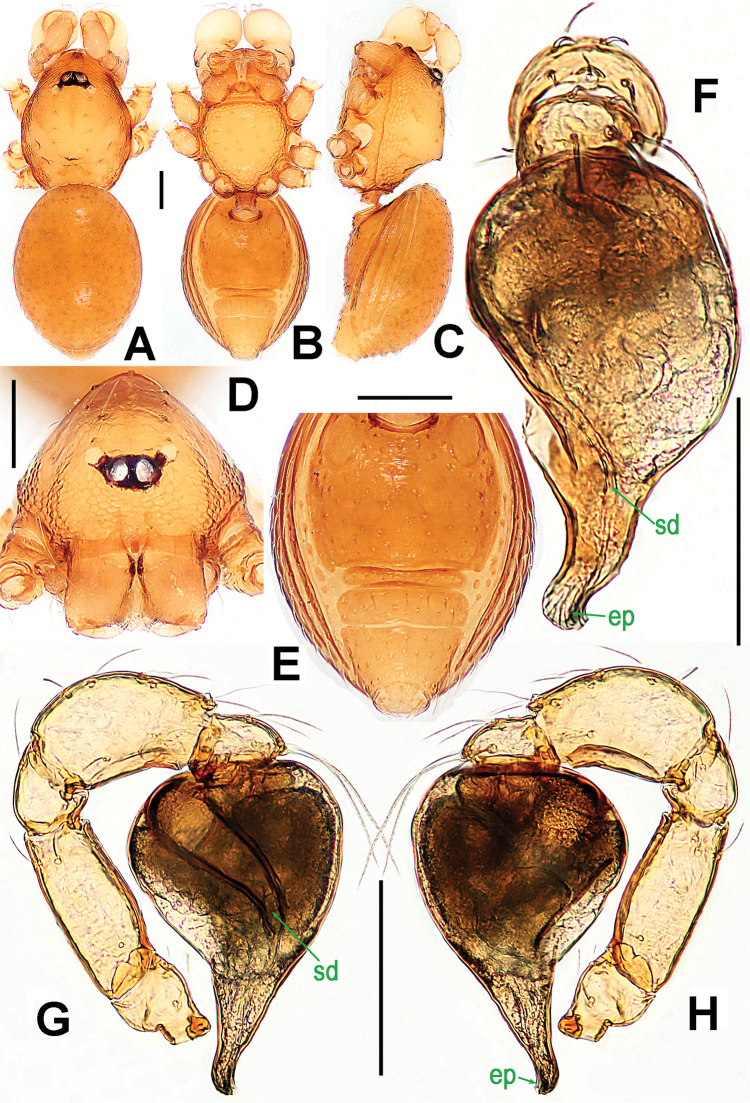
*Sulaimania brevis* sp. n., male holotype. **A–C** habitus **D** prosoma **E** opisthosoma **F–H** left palp. **A** dorsal **B, E** ventral **C** lateral **D, F** anterior **G** prolateral **H** retrolateral. Abbreviations: ep = embolic part of apes of palpal organ; sd = spermatic duct. Scale bars: 0.10 mm.

##### Other material examined.

2♂ (NHMSU), SINGAPORE: Bukit Timah Nature Reserve, Jungle Fall Stream, altitude 118 m, N1°21'25.4", E103°46'25.3", 21 August 2015, S. Li and Y. Tong leg.

##### Other species studied for comparison.

**Figure 28.**
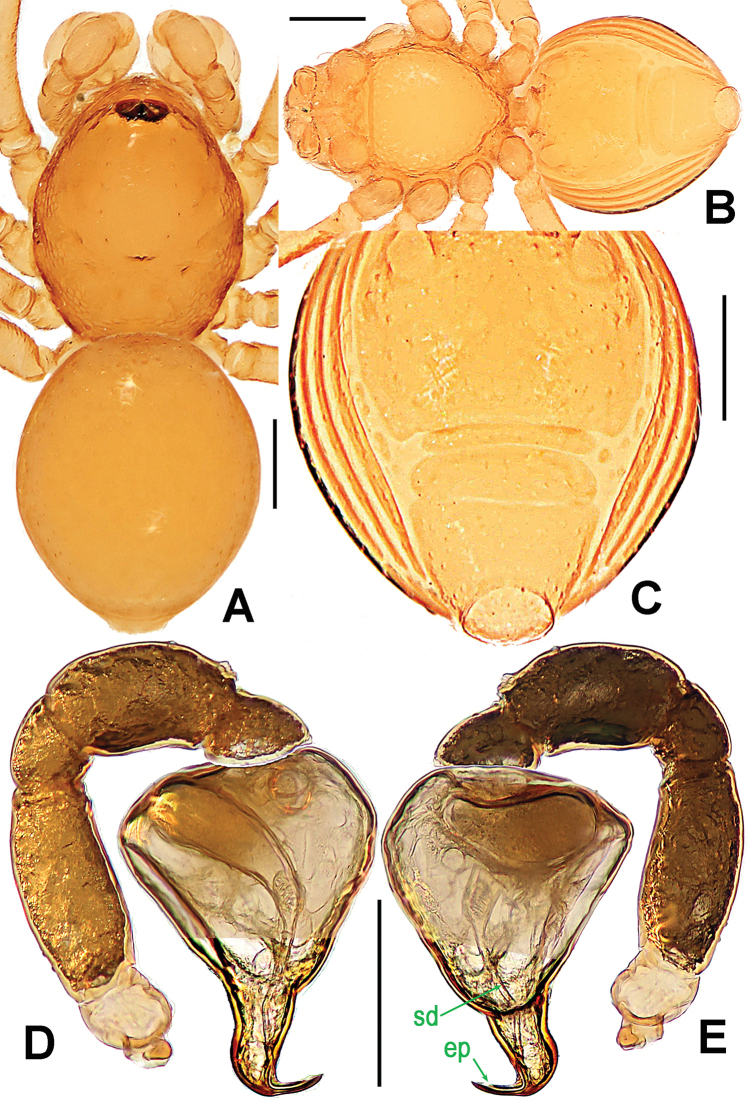
*Sulaimania vigelandi* Lehtinen, 1981, male paratype. **A–B** habitus **C** opisthosoma **D–E** left palp. **A** dorsal **B–C** ventral **D** prolateral **E** retrolateral. Abbreviations: ep = embolic part of apes of palpal organ; sd = spermatic duct. Scale bars: 0.10 mm.

*Sulaimania vigelandi* (Fig. 28A–E; Lehtinen, 1981: 51, figs 126–130, 133). Paratype 1♂ (ZMUT), MALAYSIA: Johor, Kota Tinggi District, Jalan Lombong, Biological Field Station, in litter of rain forest, 31 October to 4 November 1976, P.T. Lehtinen leg.

##### Etymology.

The specific epithet derives from the Latin word “*brevis*” = short, and refers to the short embolus; adjective.

##### Diagnosis.

This new species shares certain characteristics of the genus *Sulaimania*, including eye arrangement, habitus, and the general configuration of the male palp similar to that of the type species, *Sulaimania vigelandi* (see Fig. 28D–E and Lehtinen 1981: 51, figs 126–130, 133). However, it may be distinguished from *Sulaimania vigelandi* by the shorter embolus without a bent embolic end (Fig. 27F–H vs. Fig. 28D–E and Lehtinen 1981: fig. 129), the larger body size, the oblong carapace (Fig. 22A vs. Fig. 28A and Lehtinen 1981: fig. 127), and the sternum with reticular margins (Fig. 22B vs. Fig. 28B).

##### Description.

**Male** (holotype). Coloration: body and legs light brownish yellow.

Measurements: total length 0.78; carapace 0.46 long, 0.31 wide, 0.27 high; abdomen 0.48 long, 0.34 wide, 0.28 high; clypeus 0.10 high; sternum 0.23 long, 0.22 wide. Length of legs: I 0.78 (0.25, 0.10, 0.18, 0.12, 0.13); II 0.71 (0.22, 0.09, 0.15, 0.12, 0.13); III 0.61 (0.18, 0.08, 0.12, 0.11, 0.12); IV 0.80 (0.25, 0.08, 0.19, 0.14, 0.14). Leg formula: IV-I-II-III.

Prosoma (Fig. 27A–D): finely reticulated at clypeus and thoracic margin; cephalic area flat and smooth, bearing several long setae; AME with black base, larger than PLE in size; clypeus sharply sloping anteriorly; sternum reticulated, marginally rugose, centrally smooth, covered with sparse long setae. Legs: cuticle striated; all tibiae with 3 trichobothria, but only one at each metatarsus.

Opisthosoma (Fig. 27A–C, E): dorsal scutum long, oval, bearing short setae, anterior edge and center granulated; ventral scutum sparsely granulated, margin rugose; lateral scutum I short, perigenital scutum broad.

Palp (Fig. 27F–H): femoral cuticle faintly striated, approximately 2.5 times as long as patella; patella short, length equal to width; tibia swollen, 2 times as long as patella, 1.5 times as wide as femur, with a dorsal-distal trichobothrium; bulb inverted pyriform, surface smooth; spermatic duct looming and spiral, basally wide, and gradually tapering to embolic tip (Fig. 22F–G); embolus short, weakly sclerotized, starting from bulbous apex; embolic tip truncated, slight curving (Fig. 22F–H).

**Female.** Unknown.

##### Distribution.

Singapore.

## Supplementary Material

XML Treatment for Paculla

XML Treatment for Paculla bukittimahensis

XML Treatment for Paculla globosa

XML Treatment for Ablemma

XML Treatment for Ablemma malacca

XML Treatment for Brignoliella

XML Treatment for Brignoliella besutensis

XML Treatment for Brignoliella michaeli

XML Treatment for Singaporemma

XML Treatment for Singaporemma lenachanae

XML Treatment for Singaporemma singulare

XML Treatment for Sulaimania

XML Treatment for Sulaimania brevis

## References

[B1] Bourne JD (1981) Two new armoured spiders of the genus *Paculla* Simon, 1887 from Sarawak (Araneae: Pacullidae). Bulletin of the British Arachnological Society 5: 217–220.

[B2] Brignoli PM (1973) Ragni della Melanesia, I. Un nuovo *Tetrablemma* di Guadalcanal (Isole Salomone) (Araneae Tetrablemmidae). Memorie della Società Entomologica Italiana, Genova 52: 79–88.

[B3] Burger M (2008) Two new species of armoured spiders from Malaysia and Australia (Arachnida: Araneae: Tetrablemmidae). Bulletin of the British Arachnological Society 14: 253–261. https://doi.org/10.13156/arac.2011.14.6.253

[B4] Khmelik VV Kozub D Glazunov A (2006) Helicon Focus 3.10.3. http://www.heliconsoft.com/heliconfocus.html [accessed 22 September 2016]

[B5] Lehtinen PT (1981) Spiders of the Oriental-Australian region. III. Tetrablemmidae, with a world revision. Acta Zoologica Fennica 162: 1–151.

[B6] Levi HW (1968) The spider family Hadrotarsidae and the genus *Hadrotarsus*. Transactions of the American Microscopical Society 87: 141–145.

[B7] Levi HW Levi LR (1962) The genera of the spider family Theridiidae. Bulletin of the Museum of Comparative Zoology at Harvard College 127: 1–71.

[B8] Lin Y Li S (2010) New armored spiders of the family Tetrablemmidae from China. Zootaxa 2440: 18–32.

[B9] Lin Y Li S (2014) New cave-dwelling armored spiders (Araneae, Tetrablemmidae) from Southwest China. ZooKeys 388: 35–67. https://doi.org/10.3897/zookeys.388.5735 10.3897/zookeys.388.5735 PMC3978915 24715768

[B10] Lin Y Li S Jäger P (2012) Two new species of the family Tetrablemmidae (Araneae) from Laos and Malaysia. Zootaxa 3475: 55–64.

[B11] Murphy F Murphy J (2000) An introduction to the Spiders of Southeast Asia. Malaysian Nature Society, Kuala Lumpur, 625 pp.

[B12] Pickard-Cambridge O (1873) On some new genera and species of Araneida. Proceedings of the Zoological Society of London 41(1): 112–129. [Pl. XII–XIV]

[B13] Roewer CF (1963) Über einige neue Arachniden (Opiliones und Araneae) der orientalischen und australischen Region. Senckenbergiana Biologica 44: 223–230.

[B14] Schwendinger PJ Košulič O (2015) Two new species of armoured spiders from Vietnam and Cambodia (Araneae: Tetrablemmidae: Pacullinae). Revue Suisse de Zoologie 122(2): 423–436.

[B15] Shear WA (1978) Taxonomic notes on the armored spiders of the families Tetrablemmidae and Pacullidae. American Museum Novitates 2650: 1–46.

[B16] Simon E (1889) Etudes arachnologiques. 21e Mémoire. XXXII. Descriptions d’espèces et the genres nouveaux de Nouvelle Calédonie. Annales de la Société Entomologique de France 8(6): 237–247.

[B17] Simon E (1894) Histoire naturelle des araignées. Vol. 1, Paris, 489–760.

[B18] Thorell T (1881) Studi sui Ragni Malesi e Papuani. III. Ragni dell'Austro Malesia e del Capo York, conservati nel Museo civico di storia naturale di Genova. Annali del Museo Civico di Storia Naturale di Genova 17: 1–727.

[B19] Thorell T (1898) Viaggio di Leonardo Fea in Birmania e regioni vicine. LXXX. Secondo saggio sui Ragni birmani. II. Retitelariae et Orbitelariae. Annali del Museo Civico di Storia Naturale di Genova (2) 19[=39]: 271–378.

[B20] Tong Y (2013) Haplogynae Spiders from Hainan, China. Science Press, Beijing, 96 pp. [81 pl.]

[B21] Wheeler WH Coddington JA Crowley LM Dimitrov D Goloboff PA Griswold CE Hormiga G Prendini L Ramírez MJ Sierwald P Almeida-Silva LM Álvarez-Padilla F Arnedo MA Benavides Silva LR Benjamin SP Bond JE Grismado CJ Hasan E Hedin M Izquierdo MA Labarque FM Ledford J Lopardo L Maddison WP Miller JA Piacentini LN Platnick NI Polotow D Silva-Dávila D Scharff N Szűts T Ubick D Vink CJ Wood HM Zhang JX (2016) The spider tree of life: phylogeny of Araneae based on target-gene analyses from an extensive taxon sampling. Cladistics 2016(online version): 1–43. https://doi.org/10.1111/cla.12182 10.1111/cla.12182 34724759

